# Dynamic modeling of syngas fermentation in a continuous stirred‐tank reactor: Multi‐response parameter estimation and process optimization

**DOI:** 10.1002/bit.27108

**Published:** 2019-07-24

**Authors:** Elisa M. de Medeiros, John A. Posada, Henk Noorman, Rubens Maciel Filho

**Affiliations:** ^1^ Department of Biotechnology Delft University of Technology Delft The Netherlands; ^2^ Laboratory of Optimization, Design and Advanced Control (LOPCA), School of Chemical Engineering University of Campinas (UNICAMP) Campinas São Paulo Brazil; ^3^ DSM Biotechnology Center Delft The Netherlands

**Keywords:** dynamic model, ethanol, multiobjective optimization, parameter estimation, statistical analysis, syngas fermentation

## Abstract

Syngas fermentation is one of the bets for the future sustainable biobased economies due to its potential as an intermediate step in the conversion of waste carbon to ethanol fuel and other chemicals. Integrated with gasification and suitable downstream processing, it may constitute an efficient and competitive route for the valorization of various waste materials, especially if systems engineering principles are employed targeting process optimization. In this study, a dynamic multi‐response model is presented for syngas fermentation with acetogenic bacteria in a continuous stirred‐tank reactor, accounting for gas–liquid mass transfer, substrate (CO, H_2_) uptake, biomass growth and death, acetic acid reassimilation, and product selectivity. The unknown parameters were estimated from literature data using the maximum likelihood principle with a multi‐response nonlinear modeling framework and metaheuristic optimization, and model adequacy was verified with statistical analysis via generation of confidence intervals as well as parameter significance tests. The model was then used to study the effects of process conditions (gas composition, dilution rate, gas flow rates, and cell recycle) as well as the sensitivity of kinetic parameters, and multiobjective genetic algorithm was used to maximize ethanol productivity and CO conversion. It was observed that these two objectives were clearly conflicting when CO‐rich gas was used, but increasing the content of H_2_ favored higher productivities while maintaining 100% CO conversion. The maximum productivity predicted with full conversion was 2 g·L^−1^·hr^−1^ with a feed gas composition of 54% CO and 46% H_2_ and a dilution rate of 0.06 hr^−1^ with roughly 90% of cell recycle.

## Graphical abstract

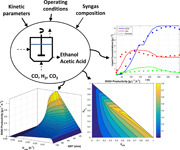

A mathematical model was developed to describe syngas fermentation in a continuous stirred tank reactor and study the effects of multiple process variables and kinetic parameters. Unknown parameters associated with substrate uptake, product selectivity, cell growth, inhibition and death were estimated using experimental data from three different publications. The authors demonstrated the potential of improving the outcomes of the process by optimizing different types of input at the same time.

## 1 INTRODUCTION

Gas fermentation is a promising biotechnological process that has gained attention due to its potential as a versatile waste‐to‐fuels route. It employs anaerobic bacteria called acetogens, which are capable of autotrophically metabolizing CO, H_2_, and CO_2_ into cell mass, acids (e.g., acetate), and solvents (e.g., ethanol and butanediol). The microbial substrate is, therefore, a gas with various possible origins; it may be, for example, (a) syngas produced via gasification of a wide range of feedstocks, including municipal solid waste and lignocellulosic biomass; (b) off‐gas from steel production and cement industries; (c) CO_2_ captured from power plants blended with H_2_ from renewable electricity, generated via electrolysis; and (d) reformed biogas (Liew et al., 2016). There has been a great expansion of gas fermentation technology over the last few years; at least three commercial‐scale ethanol plants are currently under construction (LanzaTech, 2018) or have started operation (China News Service, 2018), and many pilot plants have already been in operation for long periods of time (Liew et al., 2016). Different studies indicate that the process can play an important role in the development of a sustainable bioeconomy, being comparable to other lignocellulosic processes in terms of cost, energy efficiency, and environmental impact, while also permitting feedstock flexibility (Liew et al., 2016; de Medeiros, Posada, Noorman, Osseweijer, & Filho, 2017; Pardo‐Planas, Atiyeh, Phillips, Aichele, & Mohammad, 2017; Roy, Dutta, & Deen, 2015). From the point of view of process systems engineering, however, there is still vast room for improvement, from strain enhancement and efficient product separation, to the integrated optimization of process parameters. With that in mind, in this article, we address specifically the syngas fermentation bioreactor, coveting the presentation and analysis of a model that can be useful in optimization frameworks.

Models to describe syngas fermentation are still scarce in the literature, and only a few authors have attempted to adjust kinetic expressions to experimental data. Younesi, Najafpour, and Mohamed (2005) and Mohammadi, Mohamed, Najafpour, Younesi, and Uzir (2014) adjusted logistic curves to the growth of *Clostridium ljungdahlii* on artificial syngas using experimental data from batch fermentation essays in serum bottles. Mohammadi et al. (2014) were also able to fit Gompertz equations to their experimental profiles of product formation, and uptake rate equations for CO, presenting estimations of kinetic parameters that were later adopted by Chen, Gomez, Höffner, Barton, and Henson (2015) in their dynamic Flux Balance Analysis (FBA) model of a syngas fermentation bubble column. The latter was the first application of FBA in a dynamic model for syngas fermentation and the first spatiotemporal model of this process, but it was not compared with experimental data. The same group also published an improved version of their model, applied for CO fermentation with *Clostridium autoethanogenum* and considering uptake parameters obtained and protected by LanzaTech (Chen, Daniell, Griffin, Li, & Henson, 2018). Furthermore, Jang, Yasin, Park, Lovitt, and Chang (2017) simulated CO fermentation in a batch culture of *Eubacterium limosum* KIST612 using a dynamic model with kinetic parameters previously estimated by Chang, Kim, Lovitt, and Bang (2001), but this process results in the formation of acetic acid as the only product, which has lower a value than ethanol.

In the present study, a dynamic model was constructed for syngas fermentation with ethanol production in a continuous stirred tank reactor (CSTR). The unknown model parameters were estimated with a multi‐response minimization framework using experimental culture data from the literature and the significance of parameters was assessed with statistical analysis and generation of confidence intervals. The model was then used to study the effects of different process conditions (i.e., gas composition, dilution rate, gas residence time [GRT], and cell recycle), as well as the sensitivity of the kinetic parameters, and a multiobjective optimization was conducted for maximization of productivity and conversion. Although similar studies exist for other process, such as acetone–butanol–ethanol (ABE) fermentation (see e.g., Buehler & Mesbah, 2016), to our knowledge, there are no previous studies contemplating upon parameter estimation, statistical treatment, sensitivity analysis, and multiobjective optimization of syngas fermentation; therefore, this work was devised to fill this lacuna.

## 2 MODEL DESCRIPTION

The dynamic model developed in this study describes a stirred tank with continuous supply of syngas and batch or continuous flow of liquid, with or without cell recycle. It accounts for two phases (G/L) and seven species—CO, H_2_, CO_2_, ethanol (C_2_H_6_O or EtOH), acetic acid (C_2_H_4_O_2_ or HAc), water, and biomass–comprising 13 state variables which are the concentrations of the six chemical compounds in the gas *C*_*G,j*_ (mmol/L) and in the liquid *C*_*L,j*_ (mmol/L)—where *j* = CO, H_2_, CO_2_, EtOH, HAc, H_2_O—as well as the concentration of biomass in the liquid *C*_*X*_ (g/L). Two types of input are provided to the modeling framework (a) kinetic parameters, which define the relations between biochemical reaction rates and concentrations of chemical species and cells—these parameters are estimated in this study; and (b) operating conditions, such as gas flow rate, dilution rate, agitation rate, and syngas composition—these are specified for each of the cases analyzed in this work and their effects are further evaluated.

Fitting the model parameters with literature data turned out to be a challenge due to several reasons. First, the number of experimental papers on syngas fermentation is relatively small compared with other types of fermentation; and an even smaller number provides data without coproduction of other chemicals, such as butanol and butanediol. Among these, some provide exploratory data of very long cultures in which several accidents or interventions occur, and others fail to provide clear information about the process conditions (e.g., often the gas flow rates are omitted from the text, probably because they were not fixed during the experiment). In the present work, the model parameters were estimated for five different case studies from three different papers (C1; Phillips, Klasson, Ackerson, Clausen, & Gaddy; Phillips, Klasson, Clausen, & Gaddy, 1993); (C2; Gaddy et al., 2007); (C3A,B,C; Maddipati, Atiyeh, Bellmer, & Huhnke, 2011). These case studies have in common the use of continuous supply of syngas mixtures in stirred tanks and the formation of acetic acid and ethanol as the only products. Table 1 presents the main differences between the five scenarios, apart from the liquid medium composition, which is omitted due to space limitations. It is worth noting that C2 actually consists of 35 steady‐state points obtained under different conditions of gas composition, flow rates and agitation, while C1 and C3A,B,C comprise dynamic data. C3 is one case study subdivided in three, that is, all of the process conditions are the same, except for the concentration of yeast extract or corn steep liquor.

**Table 1:** Case studies used for the estimation of kinetic parameters

Case	C1	C2	C3A,B,C^a^
Microbe	*C. ljungdahlii*	*C. ljungdahlii*	*Clostridium* strain P11
Number of experiments under different conditions	1	35	3
Number of points per experiment (*N*_*E*_)	24	1	17
Number of types of responses (*N*_*R*_)	5	5	3
Type of data	Dynamic liquid concentrations and gas conversions	Steady‐state liquid concentrations and gas conversions	Dynamic liquid concentrations
Gas composition [H_2_:CO:CO_2_:inert]	20:55:10:15	20:65:10:5	5:20:15:60
		16:27:6:51	
		50:45:0:5	
Gas residence time (GRT; min)^b^	33–100	4.25–30	20
Dilution rate (*D*_rate_; h^−1^)	0.0035–0.012	0.018–0.083	0
Agitation [rpm]	300–450	750–900	150
Cell purge fraction (XP)^c^	0.1	0.3–1	1
Reference	Phillips et al. (1993)	Gaddy et al. (2007)	Maddipati et al. (2011)

^a^ Cases C3A to C3C differ in the amount of yeast extract (YE) or corn steep liquor (CSE), respectively, 1 g/L YE, 10 g/L CSE, and 20 g/L CSE.

^b^ Liquid volume divided by inlet gas flow rate.

^c^ Fraction of cells that are not recycled to the reactor vessel (i.e., XP = 1 when there is no cell recycle).

The next three subsections present the modeling approach for the specific production/consumption rates of species due to cell fermentation (Reaction rates); the mass balance equations considering in/out flows, gas–liquid mass transfer, fermentation, and cell recycle (Mass balance equations); and the calculation of special terms that appear in the mass balance equations (Calculation of special terms).

### 2.1 Reaction rates

**Figure 1:**
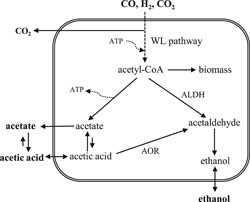
Schematic representation of syngas fermentation metabolism in *Clostridium ljungdahlii* under acidic pH, including acetic acid diffusion through the cell membrane. In this study, ethanol formation is considered possible only via the AOR pathway. ALDH, aldehyde dehydrogenase; AOR, aldehyde ferredoxin oxidoreductase

*C. ljungdahlii* and other acetogens assimilate CO, H_2_, and CO_2_ through the Wood–Ljungdahl pathway to produce acetyl‐CoA, which is then used to produce cell biomass and products, as schematized in Figure 1.

In theory, acetyl‐CoA reduction towards ethanol is possible with aldehyde dehydrogenase, but this route is always thermodynamically less favorable and actually infeasible if H_2_ is the electron donor (Bertsch & Müller, 2015). Indeed, Richter et al. (2016) found with proteome analysis of *C. ljungdahlii* that ethanol was produced exclusively through the AOR route. Ethanol production is favored when acetate accumulates inside the cell due to growth limiting conditions (i.e., biomass cannot be produced) or due to low extracellular pH (Richter et al., 2016). In the latter case, undissociated acetic acid, which is prevalent under pH lower than 4.76 (acetic acid pKa), diffuses freely through the cell membrane due to its neutral charge; however it dissociates again in the cytosol where the pH is close to neutrality and it cannot be exported through the cell membrane without active transport processes (i.e., using cellular energy), thus leading to the accumulation of acetate and protons inside the cell. In *C. ljungdahlii*, Richter et al. (2016) reported that the enzymes needed for the synthesis of ethanol were always available in excess and, as reducing equivalents are constantly being provided by the oxidation of CO and H_2_ (see Equations (1) and (2) catalyzed by carbon monoxide dehydrogenase and hydrogenase, respectively), the authors suggest that ethanol is formed as soon as undissociated acetic acid and reducing equivalents reach a threshold concentration required to make the reduction of acetic acid thermodynamically feasible.

(1) $$CO+H_{2}O\to CO_{2}+2H^{+}+2e^{-}$$

(2) $$H_{2}\to 2H^{+}+2e^{-}$$

With that in mind, we propose a kinetic model following the stoichiometry of the reactions presented in Equations (3)–(6), which intend to generally represent the chemical reactions catalyzed by the cell. The model accounts for the following assumptions: (a) The uptake rates of CO and H_2_ follow Monod kinetics with inhibition by substrate and product (Equation (7)); (b) acetic acid and ethanol inhibit substrate uptake with standard inhibition kinetics (Equation (7b)), but ethanol inhibition is only activated after a threshold concentration is achieved; (c) CO inhibits the uptake of H_2_ but not CO (Equation (7c))—this was decided after preliminary estimation routines showed that a CO inhibition constant for CO uptake could not be estimated with the experimental data used here; (d) biomass growth is a function of the uptake rates of CO and H_2_ (Equation (8)) and cell death (Equation (9)), and its composition is assumed constant; (e) acetic acid is produced from CO (Equation (3)) and H_2_/CO_2_ (Equation (4)); (f) ethanol is produced exclusively through reduction of acetic acid (Equations (5) and (6)), with reaction rates that are hyperbolic functions of the acetic acid concentration, also mimicking Michaelis–Menten kinetics (Equation (10)); (g) the effects of pH are not directly included in the model, but it is assumed that the estimated values of the kinetic parameters associated with acetic acid uptake and reduction will reflect the pH conditions adopted in the experiments used for the parameter estimation. With these assumptions, we may calculate the specific reaction rates $\nu_{k}^{R}$(mmol·g^−1^·hr^−1^)—where *k* indicates the reaction's equation number, that is, Equations (3), (4), (5), (6)—and the specific consumption/production rates of species *j*, $\nu_{j}$ (mmol.g^−1^.hr^−1^), where a negative sign in the value of $\nu_{j}$ indicates that the species is consumed, otherwise it is produced.

(3) $$4CO+2H_{2}O\to C_{2}H_{4}O_{2}+2CO_{2}$$

(4) $$4H_{2}+2CO_{2}\to C_{2}H_{4}O_{2}+2H_{2}O$$

(5) $$C_{2}H_{4}O_{2}+2CO+H_{2}O\to C_{2}H_{6}O+2CO_{2}$$

(6) $$C_{2}H_{4}O_{2}+2H_{2}\to C_{2}H_{6}O+H_{2}O$$

Ethanol inhibition in acetogens is still a research gap in the literature, but there is evidence that it occurs in a similar fashion to what is observed in the ABE fermentation, for example, Ramió‐Pujol, Ganigué, Bañeras, and Colprim (2018) observed that ethanol had inhibitory effects on *C. ljungdahlii*, though much milder than butanol, but the authors were not capable of achieving the critical concentration for full inhibition. The experimental data from case study C1 show an immediate decrease in gas conversion after the ethanol concentration surpasses 35 g/L, after which the concentrations of cells and products continue to increase for a while but eventually drop as a result of low substrate conversion. To express this behavior, the standard noncompetitive enzyme inhibition model used for ethanol inhibition is only activated after *C*_*L*,EtOH_ reaches this threshold concentration.

(7a) $$\nu_{j}=-\frac{\nu_{\max,j}\;\cdot \;C_{L,j}}{K_{S,j}+C_{L,j}}\;\cdot \;I_{E}\;\cdot \;I_{A}\;\cdot \;I_{CO,j},\;\;\;j\,=CO,H_{2}$$

(7b) $$\begin{array}{l} I_{E}=\frac{1}{1+\frac{C_{L,EtOH}}{K_{IE}}},\;\;\;\;\;\;\;I_{A}=\frac{1}{1+\frac{C_{L,HAc}}{K_{IA}}}\\ \;\; \end{array}$$

(7c) $$I_{CO\;,\;\;j=\;H_{2}}=\frac{1}{1+\frac{C_{L,CO}}{K_{I,CO}}},\;\;\;\;\;I_{CO\;,\;\;j=\;CO}=1\;$$

The specific biomass growth rate *μ* (h^−1^) is then calculated from these uptake rates via yield coefficients *Y*_*X,j*_ (g/mol) for both substrates as shown in Equation (8). Although H_2_ is not a source of carbon, it is coupled with the consumption of CO_2_ and it has also been shown to be associated with the growth rate (Mohammadi et al., 2014). The death rate *r*_*d*_ is a function of cell concentration as shown in Equation (9), where *k*_*d*_ is the death constant estimated in this study. It is worth noting that, with this equation, the growth rate is also affected by the concentration of inhibitors (ethanol, acetic acid, and CO), and the effects of other nutrients and maintenance issues are expressed in the yield coefficients and the death constant.

(8) $$\mu =-\nu_{CO}\;\cdot \;Y_{X,CO}-\nu_{H_{2}}\;\cdot \;Y_{X,H_{2}}$$

(9) $$r_{d}=k_{d}\;\cdot \;X$$

The reaction rates of acetic acid reduction (AcR), that is, $\nu_{k}^{R}$for *k* = 5 and 6, are calculated with Equations (10a) and (10b), where the parameters $\nu_{\max,j}^{AcR}$ and $K_{s,j}^{AcR}$ (*j* = CO, H_2_) are estimated in this study. The condition in Equation (10a) should be read as “for *j* = CO and *k* = 5, or for *j* = H_2_ and *k* = 6.” The expressions *F*_AcR,*j*_ are only used to make the equations clearer; they are not model parameters. The idea behind this set of equations is that acetic acid is reduced with hyperbolic kinetics limited by its concentration (Equation (10b)), and the consumption rate of CO or H_2_ necessary to provide reducing equivalents to these reactions is bounded by the total uptake rates previously calculated from Equation (7); thus it can be easily verified that $\nu_{k}^{R}\;(k=5,6)$ tends to the expression *F*_AcR,*j*_ when the uptake of CO or H_2_ is significantly larger than *F*_AcR,*j*_, whereas it tends to *−ν*_*j*_/2 when |*ν_j_*/2| is smaller than *F*_AcR,*j*_ (the division by 2 is due to the stoichiometric coefficient of CO and H_2_ in Equations (5) and (6)).

(10a) $$\nu_{k}^{R}=\left(\frac{1}{2}\right)(\frac{2F_{AcR,j}}{2F_{AcR,j}+\left|\nu_{j}\right|})\;\cdot \;\left|\nu_{j}\right|,\;\;\;\;(j,k)=(CO,5),(H_{2},6)$$

(10b) $$F_{AcR,j}=\frac{\nu_{\max,AcR}^{j}\;\cdot \;C_{L,HAc}}{K_{S,AcR}^{j}+C_{L,HAc}}\;,\;\;j=CO,H_{2}$$

The remaining substrate that is consumed can then be assumed to be used in Equations (3) and (4), and the corresponding reaction rates are calculated from Equation (11), where $\nu_{AcR,j}$ is the reaction rate of AcR (Equations (5) or (6)) using substrate *j* (i.e., CO or H_2_), for example, $\nu_{AcR,CO}$ in Equation (11) corresponds to $\nu_{5}^{R}$ as calculated from Equation (10). The total consumption/production rates of other components then follow the stoichiometry of Equations (3), (4), (5), (6) as calculated with Equations (10a), (10b), (11), (12).

(11) $$\nu_{k}^{R}=-\frac{\left(\nu_{j}+2\nu_{AcR,j}\right)}{4},\;\;\;(j,k)=(CO,3),(H_{2},4)$$

(12) $$\nu_{CO_{2}}=2\nu_{3}^{R}-2\nu_{4}^{R}+2\nu_{5}^{R}$$

(13) $$\nu_{EtOH}=\nu_{5}^{R}+\nu_{6}^{R}$$

(14) $$\nu_{HAc}=\nu_{3}^{R}+\nu_{4}^{R}-\nu_{5}^{R}-\nu_{6}^{R}$$

(15) $$\nu_{H_{2}O}=-2\nu_{3}^{R}+2\nu_{4}^{R}-\nu_{5}^{R}+\nu_{6}^{R}$$

### 2.2 Mass balance equations

The mass balance equations are presented in the following manner: the concentration fields, excepting biomass concentration, are divided into four categories regarding their phase (gas, *G* or liquid, *L*) and species type (noncondensable [NC] or condensable [C]). The governing differential equations, Equations (13), (14), (15), (16), (17), assume isothermal and isobaric operation, as well as homogeneity and constant liquid and gas volumes in the reactor.

For NC species *j* in the gas phase, $j\in \{CO,H_{2},CO_{2}\}$:

(16) $$\;\frac{dC_{G,j}}{dt}=(\frac{1}{V_{G}})\;\cdot \;\left(Q_{G,in}C_{G,j,in}-Q_{G,out}C_{G,j}\right)-k_{L}a_{j}\left(\frac{C_{G,j}}{m_{j\in NC}}-C_{L,j}\right)\left(\frac{V_{L}}{V_{G}}\right)$$

For C species *j* in the gas phase, $j\in \{EtOH,HAc,H_{2}O\}$:

(17) $$\frac{dC_{G,j}}{dt}=(\frac{1}{V_{G}})\;\cdot \;\left(Q_{G,in}C_{G,j,in}-Q_{G,out}C_{G,j}\right)+k_{L}a_{j}\left(\frac{C_{L,j}}{m_{j\in C}}-C_{G,j}\right)\left(\frac{V_{L}}{V_{G}}\right)$$

For NC species *j* in the liquid phase, $j\in \{CO,H_{2},CO_{2}\}$:

(18) $$\frac{dC_{L,j}}{dt}=(\frac{Q_{L}}{V_{L}})\;\cdot \;\left(C_{L,j,in}-C_{L,j}\right)+k_{L}a_{j}\left(\frac{C_{G,j}}{m_{j\in NC}}-C_{L,j}\right)+\nu_{j}C_{X}$$

For C species *j* in the liquid phase $j\in \{EtOH,HAc,H_{2}O\}$:

(19) $$\frac{dC_{L,j}}{dt}=(\frac{Q_{L}}{V_{L}})\;\cdot \;\left(C_{L,j,in}-C_{L,j}\right)-k_{L}a_{j}\left(\frac{C_{L,j}}{m_{j\in C}}-C_{G,j}\right)+\nu_{j}C_{X}$$

For the biomass concentration (in the liquid phase):

(20) $$\frac{dC_{X}}{dt}=(\frac{Q_{L}}{V_{L}})\;\cdot \;\left(-C_{X}\;\cdot \;XP\right)+\mu C_{X}-r_{d}$$

The gas–liquid equilibrium factors $m_{j\;\in NC}$, $m_{j\;\in C}$ in Equations (13), (14), (15), (16), (17) are described in Equations (21) and (22), where *R = *8.314 Pa·m^3^/mol·K is the ideal gas constant; MM_*L*_ and *ρ*_*L*_ refer to liquid phase molar mass (kg/mol) and density (kg/m^3^) assumed pure water at 36°C; and the respective physical parameters—Henry's law constants *H*_*j*_ (Pa), saturation pressures *P*_sat,*j*_ (Pa) and infinite‐dilution activity coefficients $\gamma_{j}^{\infty }$—can be found in the Table S1. *V*_*L*_ and *V*_*G*_ are the volumes (L) of liquid and gas inside the reactor; *Q*_*G,*in_ and *Q*_*G,*out_ are the gas volumetric flow rates (L/hr) in/out the vessel, with the latter calculated as described in Calculation of special terms; *k*_*L*_ *a*_*j*_ are mass transfer coefficients calculated as described in Calculation of special terms; *Q*_*L*_ is the liquid volumetric flow rate (L/hr). The specific rates *ν*_*j*_, *μ*, and *r*_*d*_ were presented in Reaction rates and are calculated accordingly at each time point; subscript _in_ refers to inlet gas and liquid concentrations; and XP is the cell purge fraction, that is, the fraction of cells that are not recycled to the vessel.

(21) $$m_{j\in NC}=\frac{H_{j}MM_{L}}{RT\rho_{L}}$$

(22) $$m_{j\in C}=\frac{\rho_{L}RT}{MM_{L}\gamma_{j}P_{sat,j}}$$

### 2.3 Calculation of special terms

Certain terms that appear in the right‐hand side of the ordinary differential equations (ODEs), but which are not state variables, are calculated as explained in the following.

#### 2.3.1 Outlet volumetric gas flow rate *Q*_*G*,out_

*Q*_*G,*out_ is calculated from a mole balance in the gas phase considering isobaric conditions inside the vessel. Taking into account the mass transfer of NC species (*j ∈ *NC) from gas to liquid and the mass transfer of C species (*j ∈* C) from liquid to gas, the total gas mole flow rate leaving the reactor is calculated at each time with Equation (23). *Q*_*G,*out_ is then calculated with the assumption of ideal gas in Equation (24).

(23) $$N_{G,out}\;\left(\frac{mol}{hr}\right)=Q_{G,in}\sum_{j}C_{G,j,in}-\sum_{j\in NC}\left(k_{L}a_{j}\left(\frac{C_{G,j}}{m_{j\in NC}}-C_{L,j}\right)V_{L}\right)+\sum_{j\in C}\left(k_{L}a_{j}\left(\frac{C_{L,j}}{m_{j\in C}}-C_{G,j}\right)V_{L}\right)$$

(24) $$Q_{G,out}\;\left(\frac{m^{3}}{hr}\right)=\frac{N_{G,out}RT}{P}$$

#### 2.3.2 Mass transfer coefficients

The mass transfer coefficient *k*_*L*_ *a* for air in water at *T* = 36°C is calculated via Equations (25)‐(27). It considers a weighted average between the values of *k*_*L*_ *a* estimated at 20°C for noncoalescing ($k_{L}a_{0}^{\left(20\right)}$) and coalescing ($k_{L}a_{1}^{\left(20\right)}$) broth according to the correlations proposed by van’t Riet (1979) for air in water (Equations (25c) and (25d)), where *P*_*g*_/*V*_*L*_ is the impeller power per unit volume, which is estimated from the impeller ungassed power *P*_ug_ (Equation (26)) and the correlation for the ratio *P*_*g*_/*P*_ug_ in Equations (27) (Cui, Van der Lans, & Luyben, 1996). The weighting factor *f*_*0*_ is an unknown parameter which is estimated in this study. In Equations (25)‐(27), all variables are in SI units, except for the temperature which is in °C. The ungassed power number is assumed to be *N*_*p*_ **= 12.4 for two impellers (cases C1 and C2) or *N*_*p*_ **= 16.5 (case C3) for three impellers based on the equation available in the New Brunswick Bioflo manual; *N* is the agitation rate in s^−1^; *u*_*s*_ is the gas superficial velocity (volumetric gas flow at the inlet divided by the reactor cross sectional area). In all cases, the reactor is assumed to have a height/diameter ratio of 2 and an impeller diameter of 40% the reactor diameter, as standard in New Brunswick Bioflo bioreactors.

(25a) $$\frac{k_{L}a^{\left(20\right)}}{k_{L}a^{\left(T\right)}}=1.024^{\left(20-T\right)},\;\;\;\;T=36$$

(25b) $$k_{L}a^{(20)\;}\;\;\left({hr}^{-1}\right)=f_{0}\cdot k_{L}a_{0}^{\left(20\right)}+(1-f_{0})\cdot k_{L}a_{1}^{\left(20\right)}$$

(25c) $$k_{L}a_{0}^{\left(20\right)}\;\left({hr}^{-1}\right)=3600\cdot \left(0.002{\left(\frac{P_{g}}{V_{L}}\right)}^{0.7}{\left(u_{s}\right)}^{0.2}\right)$$

(25d) $$k_{L}a_{1}^{\left(20\right)}\;\left({hr}^{-1}\right)=3600\cdot \left(0.026{\left(\frac{P_{g}}{V_{L}}\right)}^{0.4}{\left(u_{s}\right)}^{0.5}\right)$$

(26) $$P_{ug}=N_{p}\rho_{L}N^{3}{d_{i}}^{5}$$

(27a) $$\frac{Q_{G,in}\cdot N^{0.25}}{{d_{i}}^{2}}\leq 0.055,\;\;\left(1-\frac{P_{g}}{P_{ug}}\right)=9.9\left(\frac{Q_{G,in}\cdot N^{0.25}}{{d_{i}}^{2}}\right)$$

(27b) $$\frac{Q_{G,in}\cdot N^{0.25}}{{d_{i}}^{2}}\geq 0.055,\;\;\left(1-\frac{P_{g}}{P_{ug}}\right)=0.52+0.62\left(\frac{Q_{G,in}\cdot N^{0.25}}{{d_{i}}^{2}}\right)$$

The individual *k*_*L*_ *a*_*j*_ for each species is then obtained from the reference air‐water *k*_*L*_ *a* by applying the penetration theory as in Equation (28) (Talbot, Gortares, Lencki, & de la Nouë, 1991), where *Df*_*j*_ is the mass diffusivity of species *j* in water (Table S1).

(28) $$k_{L}a_{j}=k_{L}a{\left(\frac{Df_{j}}{Df_{air}}\right)}^{1/2}$$

## 3 NUMERICAL METHODS

The dynamic fermentation model described by the ODEs, Equations (16)–(20), and its supplemental algebraic equations in Model description represent a nonlinear algebraic‐differential system which demands specialized numerical solvers for stiff problems. In the present case, the *ode15s* variable‐order method from MATLAB was used for time integration from a feasible initial condition, given the appropriate value of the vector of model parameters in Equation (29). The *β* vector of parameters (*N*_*P*_ *x 1,N*_*P*_ **= 15) comprises the 14 kinetic parameters explained in Reaction rates, as well as the *k*_*L*_ *a* weighting factor *f*_*0*_

(29) $${\underline{\beta }}^{T}\equiv \left[\nu_{\max,CO}\;\;\nu_{\max,H_{2}}\;\;K_{S,CO}\;\;K_{S,H_{2}}\;\;K_{IE}\;\;K_{IA}\;\;K_{I,CO}\;\;Y_{X,CO}\;\;Y_{X,H_{2}}\;\;\nu_{\max,CO}^{AcR}\;\;K_{S,CO}^{AcR}\;\;\nu_{\max,H_{2}}^{AcR}\;\;K_{S,H_{2}}^{AcR}\;\;k_{d}\;\;\;f_{0}\right]$$

### 3.1 Estimation of model parameters

The unknown model parameters *β* were estimated as $\underline{\hat{\beta }}$ using the maximum likelihood principle (MLP; Himmelblau, 1970), with the experimental data from the case studies presented in Table 1, which are structured into five categories of response (for C1 and C2, *j* = 1 … *N*_*R*_ with *N*_*R*_ = 5) or three categories of response (for C3, *j* = 1 … *N*_*R*_ with *N*_*R*_ = 3): ethanol (*C*_*L,*EtOH_), acetic acid (*C*_*L,*HAc_), and biomass (*C*_*X*_) liquid phase concentrations (g/L), as well as CO and H_2_ conversions (*X*_CO_ and $X_{H_{2}}$ [%]), which indirectly provide information about the concentrations of these species. For C3A,B,C the gas conversions were not available, so only the liquid concentrations were used. The MLP is built with three assumptions: (A1) independency of *N*_*E*_ experiments (*i = 1 … N*_*E*_); (A2) the model is correct; and (A3) experimental responses (*y*_*j,i*_) are uncorrelated and follow normal probability density functions (PDFs) around unknown correct responses (*η*_*j,i*_) according to the variance model in Equation (30), where *r*_*j,i*_ are known response‐experiment factors and $\sigma_{\varepsilon }^{2}$ is the unknown fundamental variance (Himmelblau, 1970).

(30) $$y_{j,i}\to N\left(\eta_{j,i},\sigma_{j,i}^{2}\right),\;\;\;\;\sigma_{j,i}^{2}=r_{j,i}\sigma_{\varepsilon }^{2}$$

With Equation (30) and assumptions (A1) and (A3), it can be shown that the identities in Equations (31) result for ${\underline{y}}_{j}$, the *N*_*E*_ *× 1* vector of experimental values of response *j* at all points, where *Ε*(.), $\underline{\underline{Cov}}(.)$, ${\underline{\underline{W}}}_{j}$, and ${\underline{\eta }}_{j}$ represent, respectively, the expectancy operator, the variance‐covariance matrix operator, the *N*_*E*_ *× N*_*E*_ diagonal weight matrix for response *j* and the *N*_*E*_ *× 1* vector of correct values for response *j*.

(31a) $$Ε({\underline{y}}_{j})={\underline{\eta }}_{j}$$

(31b) $$\underline{\underline{Cov}}({\underline{y}}_{j})=\sigma_{\varepsilon }^{2}\cdot {\underline{\underline{W}}}_{j}^{-1}$$

(31c) $${\underline{\underline{W}}}_{j}^{-1}=\underline{\underline{Diag}}\left(r_{j,1},r_{j,2},\ldots ,r_{j,{\;N}_{E}}\right)$$

It can also be shown (Himmelblau, 1970) with assumptions (A1), (A2), and (A3), and Equations (30)–(31) that the application of the MLP to this multi‐response (*N*_*R*_ = 5 or 3) estimation problem results in the minimization of the weighted sums of squares of residuals written in Equation (32), where $\underline{\hat{\beta }}$ is the *N*_*P*_ *x 1* vector of estimated parameters and ${\underline{\hat{y}}}_{j}\left(\underline{\hat{\beta }}\right)$ is the corresponding *N*_*E*_ *x 1* vector of model predicted responses. Due to its high nonlinearity and likely multimodal nature, the objective function was minimized using the metaheuristic method genetic algorithm (ga MATLAB function), but a bounded simplex algorithm (fminsearch MATLAB function) was also applied to deepen a candidate optimum when a good estimate of initial point was known. In both cases, sensible lower and upper bounds were stipulated for $\underline{\hat{\beta }}$. These bounds are displayed in Table S2, jointly with ad hoc variable transformations to convert the original unrestricted simplex algorithm into a bounded simplex algorithm.

(32) $$\begin{array}{c} Min\;\;\sum_{j=1}^{N_{R}}\psi_{j}\left(\underline{\hat{\beta }}\right)\\ \{\hat{\beta }\;\}\;\;\;\;\;\;\;\;\;\;\;\;\;\;\;\; \end{array},\;\psi_{j}\left(\underline{\hat{\beta }}\right)={\left({\underline{y}}_{j}-{\underline{\hat{y}}}_{j}\right)}^{T}{\underline{\underline{W}}}_{j}\left({\underline{y}}_{j}-{\underline{\hat{y}}}_{j}\right),\;j=1...N_{R}$$

The factors $r_{j,i}$ (*j = 1…N*_*R*_, *i = 1…N*_*E*_) of the variance model of experimental responses in Equations (30) and (31), were chosen considering plausible variances of experimental values—for example, (10% of value)^2^—as well as the interests of the modeling framework, which can privilege more adherence onto some experimental responses (e.g., ethanol concentration) in detriment of others (e.g., acetic acid concentration). The underlying fact is seen in Equation (31c): as $r_{j,i}$ decrease the respective elements of the weight matrix ${\underline{\underline{W}}}_{j}$ rise, increasing the “pressure” for adherence of ${\underline{\hat{y}}}_{j}$ onto ${\underline{y}}_{j}$. In this regard, the following choices were made after multiple estimation test runs (a) for ethanol liquid concentrations (*j = 1*) $r_{j,i}={\left(0.025\cdot y_{j,i}\right)}^{2}$; (b) for acetic acid liquid concentrations (*j = 2*) $r_{j,i}={\left(0.05\cdot y_{j,i}\right)}^{2}$; (c) for biomass concentrations (*j = 3*) $r_{j,i}={\left(0.05\cdot y_{j,i}\right)}^{2}$; (d) for CO conversions *(j = 4)* $r_{j,i}={\left(0.1\cdot y_{j,i}\right)}^{2}$; and (e) for H_2_ conversions *(j = 5)* $r_{j,i}={\left(0.1\cdot y_{j,i}\right)}^{2}$.

The experimental response values were read from the dynamic profiles (C1 and C3) or steady‐state outcomes (C2) reported in the case studies considered here (see Table 1). For C1 and C3, the predicted responses ${\underline{\hat{y}}}_{j}\left(t_{i},\underline{\hat{\beta }}\right)$ at each time (with *i* = 1…N_E_) were obtained via *ode15s* numerical integration starting from the initial conditions of liquid composition reported in the respective papers; for C2, the predicted responses ${\underline{\hat{y}}}_{j}\left(i,\underline{\hat{\beta }}\right)$ were obtained with the integration starting from arbitrary initial conditions until sufficient time to reach steady state (as explained further in Steady‐state sensitivity and multi‐objective optimization the steady state was found to be nonsensitive to the initial conditions). In all cases, the initial gas‐phase concentrations were considered equal to the inlet gas concentrations and the liquid‐phase concentrations of NC species were considered equal to gas–liquid equilibrium concentrations.

### 3.2 Significance of parameters

The confidence intervals of the estimated parameters ${\hat{\beta }}_{k}$ were calculated with Equation (33), where *t*_1 – α/2_ is the abscissa at (1−α/2)⋅100%probability (*α* = .05) of the *t* Student PDF with $N_{R}\cdot N_{E}-N_{P}$ degrees of freedom (*N*_*P*_ = 15), and the estimated standard deviations ${\hat{\sigma }}_{{\hat{\beta }}_{k}}$are the *k*th diagonal elements of the estimated variance‐covariance matrix of the parameters. For a detailed explanation the reader is referred to the Supporting Information Materials provided with this work. The *F* test to reject the null hypothesis (i.e., parameter *β*_*k*_ is significant) with 95% probability is given (Himmelblau, 1970) in Equation (34), where $\phi_{1-\alpha }\left(1,N_{R}\cdot N_{E}-N_{P}\right)$ is the abscissa at (1−α)⋅100% probability (*α *= .05) of the Fisher PDF with degrees of freedom (1,$N_{R}\cdot N_{E}-N_{P}$).

(33) $${\hat{\beta }}_{k}-t_{1-\alpha /2}\;\cdot \;{\hat{\sigma }}_{{\hat{\beta }}_{k}}\;\;\;\leq \;\;\;\beta_{k}\;\;\;\leq \;\;\;{\hat{\beta }}_{k}+t_{1-\alpha /2}\;\cdot \;{\hat{\sigma }}_{{\hat{\beta }}_{k}},\;\;\;\;\;\;\;\;{\hat{\sigma }}_{{\hat{\beta }}_{k}}=\sqrt{{\left[\underline{\underline{C\overset{⌢}{o}v}}(\underline{\hat{\beta }})\right]}_{kk}}\;$$

(34) $$R({\hat{\beta }}_{k})=\frac{{\left({\hat{\beta }}_{k}\right)}^{2}}{{\left({\hat{\sigma }}_{{\hat{\beta }}_{k}}\right)}^{2}}\;\;\;>\;\;\phi_{1-\alpha }\left(1,N_{R}\;\cdot \;N_{E}-N_{P}\right)\;\;\;\;\Rightarrow \;\;\;\beta_{k}\neq 0\;\;\;\;\;\;$$

### 3.3 Steady‐state sensitivity and multiobjective optimization

After the estimation of kinetic parameters, the model was used to study the effects of several process conditions on the steady‐state productivity of ethanol (i.e., *C*_*L,*EtOH_ *∙ D*_rate_). The steady states were obtained by integrating the ODE system until all the state variables showed absolute gradients smaller than 10^−6^. This procedure was found to be faster than solving the system of nonlinear algebraic equations, as this required the initial guesses to be very close to the actual solutions. It can also be shown that, for a wide range of initial conditions, the steady state was stable and independent of such specifications (phase‐portraits depicting the dynamic trajectories are presented in Figure S2), therefore an arbitrary set of initial conditions equal to those of case study C1 was used. With this framework, the sensitivity was analyzed with respect to the gas composition (varying the molar fractions of CO and H_2_), the GRT, the dilution rate (*D*_rate_), and also to the kinetic parameters under different conditions of GRT and *D*_rate_. On the basis of these results, the process was optimized using multiobjective genetic algorithm for the maximization of two conflicting objectives: Ethanol productivity and CO conversion. The decision variables were three operating conditions (GRT, *D*_rate_, and XP—cell purge fraction) and nine kinetic parameters, which could possibly be tuned with the design of the nutrient medium, the choice of strain and/or genetic engineering. In a last study, the H_2_:CO ratio was also included as a decision variable. For this optimization routine, the bounds were specified based on the ranges of kinetic parameters estimated for the five case studies (see Table S3).

## 4 RESULTS AND DISCUSSION

### 4.1 Parameter estimation and confidence intervals

The full parameter vector $\underline{\beta }$ in Equation (29) was first estimated with the data from C1. Since C2 employed the same strain, the maximum uptake rates (*ν*_max_), saturation constants (*K*_*s*_), and inhibition constants (*K*_*I*_) were fixed and the remaining eight parameters were re‐estimated with the steady‐state data from C2. It should also be said that the ethanol inhibition term was excluded from this case study since the reported data did not achieve the threshold concentration considered in the model. Similarly, for the three case studies C3A,B,C it was considered that the difference in nutritional supplement would affect the cell yield, product selectivity, death/maintenance and the degree of coalescence of the liquid; therefore $\underline{\beta }$ was estimated for C3A (without ethanol inhibition) and for C3B and C3C all parameters were fixed except for $Y_{CO,X}$, $\nu_{\max,AcR}^{CO}$, $K_{S,AcR}^{CO}$, $k_{d}$, and *f*_*0*_, which were re‐estimated. The results are presented in Table 2 along with their 95% confidence intervals. Results of *F* test score for significance of parameters can be found in Table S4.

**Table 2:** Parameter estimates with their 95% confidence intervals

Parameter	Unit	C1	C2	C3A	C3B	C3C
$\nu_{\max,CO}$	mmol·g^−1^·hr^−1^	46.3 ± 5.33	46.3 ± 6.79	37.5 ± 5.28	37.5 ± 6.99	37.5 ± 2.70
$\nu_{\max,H_{2}}$	mmol·g^−1^·hr^−1^	31.6 ± 5.30	31.6 ± 8.47	29.5 ± 3.81	29.5 ± 4.02	29.5 ± 2.27
$K_{S,CO}$	mmol/L	0.0115 ± 0.000637	0.0115 ± 0.00631	0.0454 ± 0.00670	0.0454 ± 0.0112	0.0454 ± 0.00525
$K_{S,H_{2}}$	mmol/L	0.675 ± 0.0853	0.675 ± 0.235	0.718 ± 0.0732	0.718 ± 0.197	0.718 ± 0.0427
$K_{I,EtOH}$	mmol/L	217 ± 38.9	–	–	–	–
$K_{I,HAc}$	mmol/L	962 ± 127	962 ± 594	869 ± 117	869 ± 85.2	869 ± 183
$K_{I,CO}$	mmol/L	0.136 ± 0.0224	0.136 ± 0.110	0.827 ± 0.110	0.827 ± 0.179	0.827 ± 0.110
$Y_{X,CO}$	g/mol	0.754 ± 0.133	1.34 ± 0.226	1.69 ± 0.365	1.92 ± 0.463	2.41 ± 0.301
$Y_{X,H_{2}}$	g/mol	0.201 ± 0.0233	0.156 ± 0.0623	0.248 ± 0.0399	0.248 ± 0.0369	0.248 ± 0.0121
$\nu_{\max,CO}^{\text{AcR}}$	mmol·g^−1^·hr^−1^	24.2 ± 2.85	37.6 ± 17.6	13.0 ± 1.27	20.2 ± 1.98	8.581 ± 0.620
$K_{S,CO}^{\text{AcR}}$	mmol/L	388 ± 20.3	303 ± 163	223 ± 16.1	557 ± 169	483 ± 57.2
$\nu_{\max,H_{2}}^{AcR}$	mmol·g^−1^·hr^−1^	1.76 ± 0.166	22.2 ± 7.62	15.9 ± 1.73	15.9 ± 3.39	15.9 ± 1.16
$K_{S,H_{2}}^{AcR}$	mmol/L	464 ± 37.1	586 ± 287	72.7 ± 6.68	72.7 ± 12.6	72.7 ± 7.87
$k_{d}$	h^−1^	0.00697 ± 0.000297	0.00862 ± 0.00453	0.0119 ± 0.00135	0.0112 ± 0.00271	0.00959 ± 0.00163
$f_{0}$	–	0.988 ± 0.0464	0.958 ± 0.339	0.700 ± 0.0699	0.973 ± 0.243	0.988 ± 0.123

The estimated values of the maximum CO and H_2_ uptake rates ${\hat{\nu }}_{\max,CO}$ and ${\hat{\nu }}_{\max,H_{2}}$ are comparable with CO uptake rates reported by other authors: Chen et al. (2018) obtained CO uptake rates from 41 to 43 mmol·g^−1^·hr^−1^ in continuous cultures of *C. autoethanogenum* in a bubble column; Mohammadi et al. (2014) estimated a maximum rate of 34 mmol·g^−1^·hr^−1^ for *C. ljungdahlii*; and Gaddy et al. (2007) reported a large range of 14–100 mmol·g^−1^·hr^−1^ for different operating conditions with *C. ljungdahlii*. The saturation constants *K*_*s*_, which are inversely related to the microbe's affinity to the substrate, reflect the disparity observed between the conversions of CO and H_2_ in cases C1 and C2: *K*_*S,*CO_ is around 2% the value of $K_{S,H_{2}}$ in C1 and C2, and 6% in C3, while the pure component solubility of CO is only approximately 13% higher that of H_2_ at the culture temperature. With regard to the inhibition constants, it can be concluded that the effect of CO on H_2_ uptake was higher in cases C1 and C2 given the lower value of *K*_*I,*CO_ for these cases, although it should also be noted that cases C3 use a small percentage of only 5% H_2_ in the feed gas. Acetic acid inhibition was also found to be statistically significant although its large value in all cases (>850 mmol/L) indicate small inhibitory effects under the conditions of the experiments considered here. For Case C2, the uncertainty associated with this parameter is also notably high, reaching around 60% of its nominal value while in the other case studies this percentage is less than 20%. In fact, for most of the estimated parameters, C2 presents the highest uncertainties among the cases, which is also due to the large variety of experimental conditions adopted in this case and relatively small number of samples. It should be noted that as more experiments are performed, new data can be incorporated into the modeling framework presented here and the parameters can be re‐estimated with higher accuracy.

The cell yields, specifically *Y*_*X,*CO_, showed a wide variation among the five case studies, being the highest for C3C (the experiment with high concentration of corn steep liquor) at 2.41 g/mol (nominal value). As expected the value of *Y*_*X,*CO_ increases from C3A to C3C as a result of increasing concentrations of nutritional supplement. Clearly this parameter is specific to the culture conditions and microbial strain, and this can be verified by looking at the diversified results of cell yield in syngas fermentation reported by different authors, some of which are in good agreement with this study: 0.25 g/mol for clostridial bacteria P7 (Rajagopalan, Datar, & Lewis, 2002); 1.4 g/mol for *C. ljungdahlii* (Phillips, Clausen, & Gaddy, 1994); 2.1–3.2 g/mol for *C. ljungdahlii* (Mohammadi, Mohamed, Najafpour, Younesi, & Uzir, 2016); 2 g/mol for *Rhodospirillum rubrum* (Kerby, Ludden, & Roberts, 1995); 7.2 g/mol for *E. limosum* KIST612 (Chang et al., 2001).

**Figure 2:**
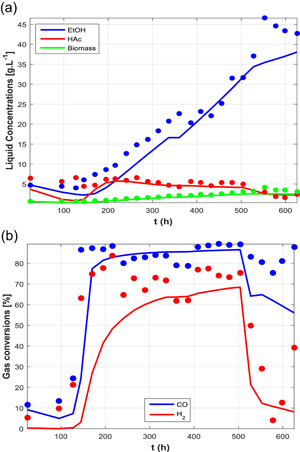
Predicted dynamic profiles (solid lines) and experimental points (circles) for case study C1. (a) Concentration of products and cells in the liquid; (b) conversions of CO and H_2_ [Color figure can be viewed at wileyonlinelibrary.com]

**Figure 3:**
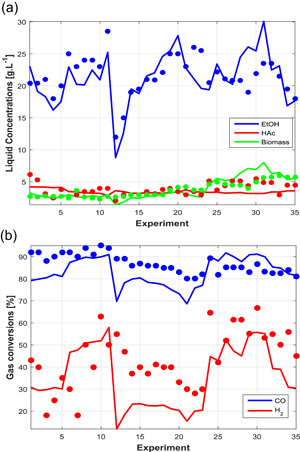
Predicted steady‐state responses (solid lines) and experimental points for case study C2. (a) Concentration of products and cells in the liquid; (B) conversions of CO and H_2_ [Color figure can be viewed at wileyonlinelibrary.com]

**Figure 4:**
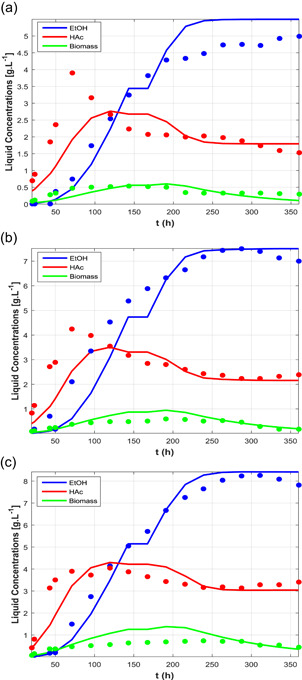
Predicted dynamic profiles (solid lines) of products and cells, and experimental points (circles) for case studies C3. (a) Case C3A; (b) Case C3B; (c) Case C3C [Color figure can be viewed at wileyonlinelibrary.com]

The results generated by the model with the different parameter vectors are shown in Figures 2, 3, 4 along with the respective experimental points. The model showed overall reasonable predictive power for ethanol, acetic acid, and biomass concentrations, although certain dynamic features were only roughly captured. For example, in cases C3 the acetic acid peak around 75 hr was flattened and slightly displaced to the right (this was also the tendency of the experimental data going from case C3A to C3C). In Case C1, the model was able to predict the conversion decrease after 500 hr, but the experimental data also suggest a recovery which could not be predicted by the model. In the modeling framework, this decrease is a consequence of joint inhibitory effects of ethanol and CO, the latter which accumulates in the liquid phase due to impaired uptake as a consequence of the former, and acetic acid to a smaller extent. Since this is the only experiment with such high concentrations of ethanol, it is unclear whether this behavior is due to product inhibition or if other external factors could be the cause of this perturbation. Although it is likely that ethanol exhibits inhibitory effects, as demonstrated by Ramió‐Pujol et al. (2018) and Férnandez‐Naveira, Abubackar, Veiga, and Kennes (2016), a final conclusion cannot be drawn from the current set of experiments with regard to this matter, and further experimental investigation is needed to evaluate critical product concentration and inhibition constants.

Case C2 (steady state) showed the highest deviations from the experimental data as well as parameter uncertainties, which is probably due to the large range of process conditions encompassed by the data. It should also be noted that it is unclear whether the medium composition was kept fixed or not during these experiments. Nonetheless, the model was still good at capturing the tendency of the data, especially the concentrations of products and cells. In comparison with C1, which used the same strain, the AcR parameters were more favorable to ethanol production, that is, with higher $\nu_{\max,j}^{AcR}(j=CO,H_{2})$. The saturation constants $K_{S,j}^{AcR}(j=CO,H_{2})$ were similar if we consider the confidence intervals.

The last parameter, *f*_*0*_, indicates the level of coalescence in the liquid, with higher *f*_*0*_ (as in cases C1 and C2) meaning the liquid is highly noncoalescing and thus enables higher gas–liquid mass transfer coefficients. It is worth noting that *f*_*0*_ increased from Case C3A to C3C and specially from C3A to C3B (when 1 g/L yeast extract was replaced with 10 g/L corn steep liquor).

### 4.2 Sensitivity of process conditions and kinetic parameters

**Figure 5:**
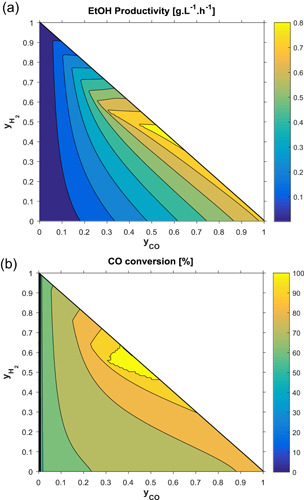
Steady‐state ethanol productivity achieved with different gas compositions ($y_{CO_{2}}=1-y_{CO}-y_{H_{2}}$) with fixed conditions: GRT = 20 min, *D*_rate_ = 0.025 hr^−1^, *N* = 500 rpm, cell recycle = 90% [Color figure can be viewed at wileyonlinelibrary.com]

With the fitted models, the performance of the bioreactor was evaluated for different conditions of gas composition, dilution rate and gas flow rates. For these sensitivity analyses, the parameter vector estimated in C2 was used as basis. The effects of syngas composition are depicted in Figure 5 for ethanol productivity and CO conversion. It can be seen that both responses are enhanced with the CO content, but there is a maximum outcome at H_2_:CO close to 1 and the peak is slightly dislocated to the left (higher H_2_:CO) for CO conversion. This result suggests that the syngas composition can be tuned to improve the performance of the bioreactor, but the optimal composition would, of course, depend on the balance between extra productivity/conversion in the bioreactor and extra energy costs in upstream operations (gasification and gas conditioning). It was also observed that cell mass concentration always increased with the fraction of CO, going from near 1 g/L at low values up to 11 g/L with pure CO (figure shown in Figure S5).

**Figure 6:**
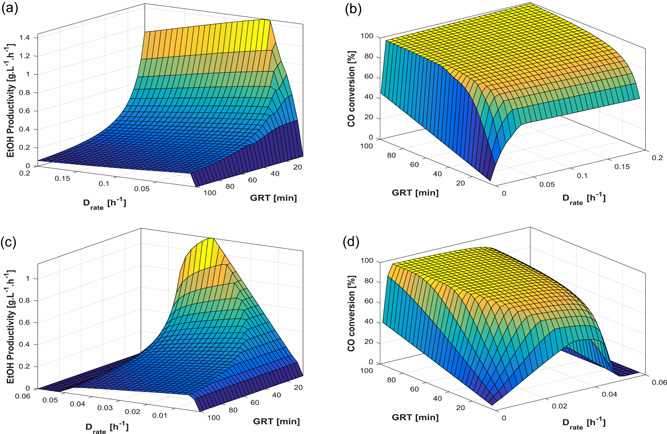
Steady‐state ethanol productivity and CO conversion as a function of gas residence time (GRT) and liquid dilution rate (*D*_rate_) (a,b) with 90% cell recycle; (c,d) without cell recycle. Fixed conditions $y_{CO}$ = 0.65, $y_{H_{2}}$ = 0.2, $y_{CO_{2}}$ = 0.15, *N* = 500 rpm. The axes are rotated in (a) and (c) [Color figure can be viewed at wileyonlinelibrary.com]

Assuming fixed gas composition of a CO‐rich gas, the response surfaces shown in Figure 6 were generated to illustrate the effects of dilution rate (*D*_rate_) and GRT with cell recycle (10% purge) and without. Both cases demonstrate how lower values of GRT (i.e., higher gas flow rates) enhance the productivity due to higher supply of substrate as well as higher gas–liquid mass transfer coefficient, although at the expense of the CO conversion. Moreover, as typically observed in chemostat cultures, the productivity is a concave function of the dilution rate with a clear maximum—in this case, also dependent on the gas flow rate. From Figure 6, it is also clear that cell recycle enhances the ethanol productivity (the maximum increases from around 1.13–1.44 g·L^−1^·hr^−1^) and broadens the region of operation without cell wash‐out. Moreover, the maximum cell mass concentration increases from 4.5 to around 16 g/L when cell recycle is used (response surfaces shown in Figure S6). The effects of agitation are not shown here, but response surfaces with this variable can be found in Figure S3. Evidently, increasing the agitation rate also enhances the mass transfer of CO and H_2_ between the gas and the liquid, which allows for higher conversions and ethanol productivity; however, at the price of higher energy consumption.

The same response surfaces were constructed for H_2_‐rich gas (see Figure S4), which had overall the same shape and tendencies as Figure 6. In accordance with Figure 5, it was also observed that increasing the content of H_2_ by adopting a gas composition of [H_2_:CO:CO_2_] = [50:45:5] increased the maximum productivity to around 1.6 g·L^−1^·hr^−1^ when cell recycle was used. However the maximum productivity under no cell recycle actually decreased from 1.13 to 0.93 g·L^−1^·hr^−1^.

**Figure 7:**
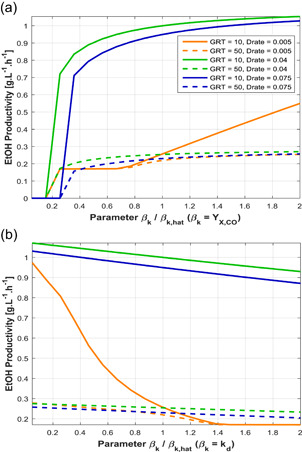
Sensitivity of steady‐state ethanol productivity to kinetic parameters under different conditions of gas residence time (GRT) and liquid dilution rate (*D*_rate_). (a) Cell yield on CO (*Y*_*X,*CO_); (b) cell death rate constant (*k*_*d*_). Fixed conditions $y_{CO}$ = 0.65, $y_{H_{2}}$ = 0.2, $y_{CO_{2}}$ = 0.15, *N* = 500 rpm. The corresponding profiles of cell mass concentration are shown in Figure S7 of the Supporting Information Material [Color figure can be viewed at wileyonlinelibrary.com]

With regard to the effects of kinetic parameters, it was observed that the operating conditions contributed significantly to the sensitivity of this type of variable. Two illustrative examples are given in Figure 7, where the parameters vary from 0.05 to 2 times their nominal value (as obtained in C2), and 6 combinations of low/high gas flow rate and dilution rate are employed. It can be seen that not only do lower values of GRT improve the productivity, but they also enhance the effects of changing the kinetic parameters (see the inclination of solid lines in Figure 7 in comparison with the dashed lines). Figure 7a also suggests that increasing *Y*_*X,*CO_ will eventually lead to the same outcome of productivity for different values of *D*_rate_ under the same GRT (see green and blue lines). Very low values of *D*_rate_, however, showcase the opposite trend: The two orange lines (*D*_rate_ = 0.005 hr^−1^) are coincident until a bifurcation occurs at $\beta_{k}/{\hat{\beta }}_{k}\approx 0.75$. These and other kinetic parameters showed considerable variation between the five estimations as presented in Parameter estimation and confidence intervals, indicating that such microbial properties can be customized with the selection of strain and medium composition, besides of course genetic engineering which would be a natural extrapolation of this conclusion. The performance of the bioreactor can therefore be improved with integrated design considering the simultaneous effects of biokinetic parameters and process conditions.

### 4.3 Optimization of ethanol productivity and CO conversion

**Figure 8:**
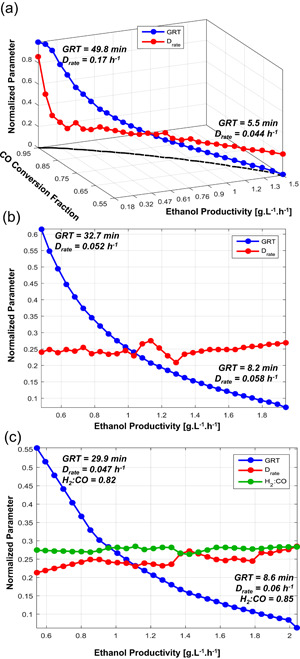
Pareto‐optimal solutions for maximization of steady‐state ethanol productivity and CO conversion (a) Fixed gas composition at 65% CO, 20% H_2_, and 15% CO_2_ with normalized decision variables GRT and *D*_rate_ plotted in the *z*‐axis; (b) fixed gas composition at 45% CO, 50% H_2_, and 5% CO_2_, all points correspond to 100% CO conversion; (c) H_2_:CO ratio free to vary between 0 and 3, all points correspond to 100% CO conversion. In all cases the decision variables are normalized with respect to their lower and upper bounds, that is, GRT between 5 and 50 min and *D*_rate_ between 0.005 and 0.2 hr^−1^. The values of the most relevant decision variables at the solutions are shown in Tables S6–S8 [Color figure can be viewed at wileyonlinelibrary.com]

The solutions to three optimization runs are shown in Figure 8. The multiobjective optimization was first solved for three operating conditions (GRT, *D*_rate_, and XP) and nine kinetic parameters (all excepting the saturation and inhibition constants, which were fixed at the values obtained for C2). The lower and upper bounds were chosen based on the intervals of the parameters estimated in Table 2 (see Table S3); for the operating conditions, the GRT was free to vary in the range 5–50 min, *D*_rate_ in the range 0.005–0.2 hr^−1^ and XP in the range 0.1–1 (this meaning no cell recycle). In the first run, the gas composition was fixed for a CO‐rich gas, that is, [H_2_:CO:CO_2_] = [20:65:15]. For this case, the Pareto‐optimal points reflected the classical problem of two conflicting objectives (Figure 8a): Higher gas flow rates (lower GRT) can enhance the productivity at the expense of CO conversion, as a higher fraction of the gas is also wasted. The Pareto front in Figure 8a is projected on the *x*–*y* axis, with the highest productivity (1.5 g·L^−1^·hr^−1^) corresponding to 55% CO conversion. These points are the so‐called nondominated solutions, at which none of the objective functions can be improved without harming the other. The decision variables GRT and *D*_rate_ tied to these points are plotted along the Pareto curve with their normalized values on the *z*‐axis. The other decision variables (including kinetic parameters) are not shown because their variation along the Pareto curve was considerably smaller, but maximum and minimum values of all decision variables from the set of Pareto‐optimal points are presented in Table S5. In a second run (Figure 8b), the gas composition was changed to a H_2_‐rich gas, that is, [H_2_:CO:CO_2_] = [50:45:5]. Unexpectedly, for this case the CO conversion could be maximized to 100% over a wide range of productivities, therefore instead of depicting a Pareto front, Figure 8b presents the solutions of ethanol productivity obtained under 100% CO conversion, with the corresponding normalized GRT and *D*_rate_ plotted on the *y*‐axis. It's also worth noting that, with a high content of H_2_, even a relatively small GRT of 8.2 min enabled full conversion of CO whereas also achieving a high productivity of 1.92 g·L^−1^·hr^−1^—this point can thus be selected as the optimal solution in terms of both productivity and conversion.

By analyzing the results of the kinetic parameters in both runs, it is clear that employing a H_2_‐rich gas also boosts the sensitivity of H_2_‐related parameters, in special the parameter $\nu_{\max,H_{2}}^{AcR}$ which is related to the reduction of acetic acid—the average value of this parameter in the optimal solutions increased from 1.4 to nearly 20 mmol·g^−1^·hr^−1^ from the CO‐rich to the H_2_‐rich optimization run. Interestingly, the parameter $\nu_{\max,CO}^{AcR}$ was on average 20% smaller in the second case, while the other parameters remained more or less constant. It was also noted that the parameters of cell yield on CO and death constant were close to their specified bounds in both cases, with *Y*_*X,*CO_ being close to 2.4 g/mol (the value estimated with the data from C3C) and *k*_*d*_ being close to 0.007 hr^−1^ (the value estimated with the data from C1). While this result demonstrates the efficacy of manipulating kinetic parameters to improve bioreactor performance, it also raises the question as to whether it would be feasible in real operation to use medium formulation and genetic engineering to change the parameters independently from each other.

Finally, a third optimization was conducted adopting the H_2_:CO ratio in the feed gas as a new decision variable, which was allowed to vary between 0 (pure CO) and 3 (25% CO and 75% H_2_). Also for this case, shown in Figure 8c, the solutions did not form a Pareto front, as 100% CO conversion was attainable for a wide range of productivities. Figure 8c is hence analogous to Figure 8b but H_2_:CO is included, although it is basically constant for all solutions at around 0.78–0.86. The maximum productivity that can be obtained with 100% CO conversion is just over 2.0 g·L^−1^·hr^−1^, which is achieved with GRT = 8.6 min, *D*_rate_ = 0.06 hr^−1^, XP = 0.11, and H_2_:CO = 0.85 (i.e., 54% CO and 46% H_2_). The kinetic parameters at this solution are: $\nu_{\max,CO}$ = 40.3 mmol·g^−1^·hr^−1^, $\nu_{\max,H_{2}}$ = 34.8 mmol·g^−1^·hr^−1^, $Y_{X,CO}$ = 2.37 g/mol, $Y_{X,H_{2}}$ = 0.223 g/mol, $\nu_{\max,CO}^{AcR}$ = 34.2 mmol·g^−1^·hr^−1^, $\nu_{\max,H_{2}}^{AcR}$ = 16.6 mmol·g^−1^·hr^−1^, $K_{S,CO}^{AcR}$ = 398 mmol/L, $K_{S,H_{2}}^{AcR}$ = 396 mmol/L, and *k*_*d*_ = 0.00546 hr^−1^. It is noteworthy that all of these parameters, with the exception of the yield coefficients, remain relatively close to the nominal parameters estimated for C2, in fact inside their confidence intervals, which suggests that efforts should be concentrated on enhancing the cell yields and not, for example, the maximum uptake rates (at least for the conditions of gas–liquid mass transfer encompassed by this study). Even though *ν*_max_ is directly associated with the cell's capacity to take up substrate, the uptake rate might just be limited by the mass transfer, such that after a certain point there would be no actual gain with increasing *ν*_max_.

Another result from the optimization studies is that high productivities would be attained with very large cell concentrations reaching up to 30 g/L (results shown in Figure S8), although this depends on the gas composition. For example, 1 g·L^−1^·hr^−1^ of ethanol productivity would be attainable with H_2_‐rich gas with operating conditions and kinetic parameters that result in 10 g/L of cell mass, while for CO‐rich gas the cell concentration would be a little over 20 g/L for the same ethanol productivity.

Agitation rate and gas recycle rate are also important operating variables which were not included in this study, but should be evaluated in the future with the inclusion of power consumption as a third objective function. It is possible, for example, that under certain conditions the gains in productivity and conversion might compensate for any extra spending with electricity. Other reactor designs should also be evaluated, such as bubble column, gas‐lift, and membrane reactors. Ultimately, however, the bioreactor should be optimized simultaneously with other unit operations, such as gasification and distillation, since optimal conditions in one unit might lead to worse outcomes in other units with respect to economic and/or environmental issues.

## 5 CONCLUSIONS

A dynamic model was presented for the production of ethanol via syngas fermentation in a CSTR, and unknown kinetic parameters were estimated with literature data employing different conditions of gas flow rate, dilution rate, syngas composition, and medium composition. The modeling framework was then used to evaluate the effects of different input variables on the outcomes of ethanol productivity and gas conversion, and it was observed that cell recycle rate, gas flow rate, and H_2_ content had clear positive effects on the productivity, while the dilution rate gives a different maximum depending on the other variables. Moreover, the kinetic parameters were found to have different sensitivity patterns depending on the process conditions, for example some of them having larger effects on the productivity when higher gas flow rates are used. Since these parameters are specific to the type of strain and composition of the liquid medium, we conducted an optimization of productivity and conversion using operating conditions and kinetic parameters as decision variables, thereby showing the possibility of attaining higher values of both responses at the same time. Implementation of the results predicted in this work would require further studies connecting the kinetic parameters to the exact aspects of the liquid medium and strain capabilities, as well as more experiments investigating the inhibitory effects of products and CO. Therefore, as more experimental data become available, the modeling framework presented here can be used to re‐estimate parameters, generate more accurate results and provide new insights for integrated process optimization.

## CONFLICT OF INTERESTS

The authors declare that there are no conflict of interests.

**NOMENCLATURE**
  

**Greek Symbols**
$\underline{\beta },\;\underline{\hat{\beta }}$
: vector of parameters: correct, estimated
$\gamma_{j}^{\infty }$
: activity coefficient at infinite dilution of component *j* in water at 36°C
${\underline{\eta }}_{j}$
: vector of correct responses *j*
*μ*
: biomass specific growth rate (h^−1^)
$\nu_{j}$
: specific production/consumption rate of component *j* (mmol·g^−1^·hr^−1^)
$\nu_{k}^{R}$
: specific rate of reaction number *k* (mmol·g^−1^·hr^−1^)
*ρ*_*L*_
: mass density of water at 36°C (kg/m^3^)
$\sigma_{\varepsilon }^{2}$
: unknown fundamental variance
$\hat{\sigma }$
: estimated standard deviation

**Roman Symbols**
*C*_*L,j*_
: concentration of component *j* in the liquid phase (mmol/L or g/L)
*C*_*G,j*_
: concentration of component *j* in the gas phase (mmol/L)
*C*_*X*_
: concentration of biomass in the liquid (g/L)
$\underline{\underline{Cov}},\underline{\underline{C\overset{⌢}{o}v}}$
: variance‐covariance matrix: correct, estimated
*D*_*f,j*_
: mass diffusivity of component *j* in water at 25°C (cm^2^/s)
*D*_rate_
: dilution rate = *Q*_*L*_ */V*_*L*_ (hr^−1^)
*d*_*i*_
: impeller diameter (m)
*f*_*0*_
: broth coalescence weighting factor
GRT: gas residence time = *V*_*L*_ */Q*_*G,in*_ (min)
*H*_*j*_
: Henry's law constant of component *j* dissolved in water at 36°C (Pa)
$k_{L}a_{0}^{\left(20\right)},k_{L}a_{1}^{\left(20\right)}$
: mass transfer coefficient of air in water at 20°C: noncoalescing and coalescing broth (hr^−1^)
$k_{l}a_{j}$
: mass transfer coefficient of component *j* in water at 36°C (hr^−1^)
MM_*L*_
: molar mass of water (kg/mol)
*N*
: agitation rate (rpm or s^−1^)
*N*_*E*_
: number of experimental points
*N*_*P*_
: number of unknown parameters
*N*_*p*_
: ungassed power number
*N*_*R*_
: number of response variables used for parameter estimation
*P*
: pressure inside reactor (Pa)
*P*_*g*_, *P*_*ug*_
: gassed power and ungassed power (W)
*P*_sat,*j*_
: vapor pressure of component *j* at 36°C (Pa)
*Q*_*G*_, *Q*_*L*_
: gas and liquid volumetric flow rates (m^3^/s, m^3^/hr, ml/hr, or ml/min)
*R*
: universal gas constant (J/mol·K)
*r*_*j,i*_
: stipulated response‐experiment factors
*T*
: temperature (°C or K)
*u*_*s*_
: superficial gas velocity (m/s)
*V*_*G*_,*V*_*L*_
: volume of gas and volume of liquid inside the reactor (ml, L, or m^3^)
${\underline{\underline{W}}}_{j}$
: weight matrix of response *j*
*X*_CO_, $X_{H_{2}}$
: gas conversions of CO and H_2_
XP: cell purge fraction
${\underline{y}}_{j},\;{\underline{\hat{y}}}_{j}$
: vector of responses (g/L): experimental, predicted

## Supporting information

Supplementary information
