# Supplementary material for: Dynamic modeling of syngas fermentation in a continuous stirred‐tank reactor: Multi‐response parameter estimation and process optimization
Source: Biotechnol Bioeng. 2019 Jul 24;116(10):2473–87. doi: 10.1002/bit.27108 (PMC9328424; doi:10.1002/bit.27108)
Supplement: Supplementary file 1 — Supplementary information [file BIT-116-2473-s001.pdf]

SUPPLEMENTARY MATERIALS

Dynamic modeling of syngas fermentation in a continuous stirred tank reactor: multi-response parameter estimation and process optimization

Elisa M. de Medeiros[1,2], John A. Posada[1], Henk Noorman[1,3], Rubens Maciel Filho[2]

[1] Department of Biotechnology, Delft University of Technology, van der Maasweg 9, 2629 HZ, Delft, The Netherlands
[2] School of Chemical Engineering, University of Campinas (UNICAMP), Av. Albert Einstein 500, 13084-852, Campinas, SP, Brazil
[3] DSM Biotechnology Center, PO Box 1, 2600 MA Delft, The Netherlands

Corresponding author

Elisa M. de Medeiros, E-mail: E.MagalhaesdeMedeiros@tudelft.nl

Lag-phase

The uptake rates of CO and H_2 were multiplied by a lag-phase term when the experimental data suggested such behavior. This was done only for the dynamic case study C1, in which the uptake rates v_j (j = CO, H_2) were multiplied by I_{lag} as described in Eq. (S1), to simulate lag-phase behavior during the first 150 hours of fermentation.

$$I_{lag} = \left(1 + \exp\left(-0.15 \cdot \left(t - 150\right)\right)\right)^{-1} \tag{S1}$$

Table S1. Species properties for gas-liquid equilibrium and mass transfer coefficients.

	CO	H_2	CO_2	EtOH	HAc	H_2O
H_j †	4.972×10^{9}	6.692×10^{9}	1.205×10^{8}	-	-	-
$P_{sat,j}$ ‡	-	-	-	1.523×10^{4}	4.047×10^{3}	6.260×10^{3}
γ_j^{∞} §	-	-	-	7.6	3.5	-
Df_j ¶	2.03×10^{-5}	4.50×10^{-5}	1.92×10^{-5}	0.84×10^{-5}	0.99×10^{-5}	-

† Henry constants (Pa) in water at 36°C calculated with the correlations reported in Sander (1999).
‡ Vapor pressure (Pa) at 36°C calculated with Antoine equation.
§ Activity coefficients at infinite dilution in water predicted with UNIFAC model using Aspen Plus.
¶ Mass diffusivity ($cm^2.s^{-1}$) in liquid water at 25°C from Cussler (1997). Since for a species i the relationship $Df_i \cdot \mu_{H_2O} / T$ is constant (Perry and Green, 1999), it can be shown that the relationship Df_i / Df_{air} is also constant with temperature. The mass diffusivity of air in water at 25°C is $2\cdot10^{-5}$ $cm^2.s^{-1}$.

Table S2. Bounds used in parameter estimation.

Parameter β_k	Unit	Lower Bound	Upper Bound
$v_{\max,CO}$	mmol.g^{-1}.h^{-1}	15	60
$v_{\max,H_2}$	mmol.g^{-1}.h^{-1}	15	60
$K_{S,CO}$	mmol.L^{-1}	0.001	0.9
K_{S,H_2}	mmol.L^{-1}	0.001	0.9
$K_{I,EtOH}$	mmol.L^{-1}	100	1000
$K_{I,HAc}$	mmol.L^{-1}	100	1000
$K_{I,CO}$	mmol.L^{-1}	0.1	1
$Y_{CO,X}$	g.mol^{-1}	0.1	3
$Y_{H_2,X}$	g.mol^{-1}	0.1	3
$v_{\max,AcR}^{CO}$	mmol.g^{-1}.h^{-1}	1	50
$K_{S,AcR}^{CO}$	mmol.L^{-1}	20	600
$v_{\max,AcR}^{H_2}$	mmol.g^{-1}.h^{-1}	1	50
$K_{S,AcR}^{H_2}$	mmol.L^{-1}	20	600
k_d	h^{-1}	0.001	0.05
f_0	-	0.1	1

Table S3. Bounds used in the optimization of ethanol productivity and CO conversion.

Parameter β_k	Unit	Lower Bound	Upper Bound
GRT	min	5	50
D_{rate}	h^{-1}	0.005	0.2
XP	-	0.1	1
$H_2{:}CO$	-	0	3
$v_{\max,CO}$	mmol.g^{-1}.h^{-1}	35	50
$v_{\max,H_2}$	mmol.g^{-1}.h^{-1}	25	40
$Y_{X,CO}$	g.mol^{-1}	0.6	2.5
Y_{X,H_2}	g.mol^{-1}	0.1	0.3
$v_{\max,AcR}^{CO}$	mmol.g^{-1}.h^{-1}	20	50
$K_{S,AcR}^{CO}$	mmol.L^{-1}	300	500
$v_{\max,AcR}^{H_2}$	mmol.g^{-1}.h^{-1}	1	30
$K_{S,AcR}^{H_2}$	mmol.L^{-1}	300	600
k_d	h^{-1}	0.005	0.01

Bounded Simplex Algorithm

As explained in the main text, the estimated parameters presented in this study were obtained using Genetic Algorithm, but the derivative-free method Simplex was also used for deepening . The MATLAB function *fminsearch* applies Simplex Algorithm for unconstrained multi-variable problems, but in the present study the parameters are constrained as shown in Table S2. In order to use this method with constraints on the optimization variables, the vector of constrained parameters being estimated $\underline{\hat{\beta}}$ must be transformed into a new vector of unconstrained parameters $\underline{\omega}$, whose individual elements ω_k are computed with Eq. (S2) from a given initial estimate $\underline{\hat{\beta}}$, where LB_k and UB_k are the lower and upper bounds pre-established for parameter $\hat{\beta}_k$.

Then, during the Simplex search, the subroutine that receives the unconstrained parameters and generates the responses $\underline{\hat{y}}_j$ in each iteration, receives the new unconstrained vector $\underline{\omega}$ and converts it back to $\underline{LB} \le \underline{\hat{\beta}} \le \underline{UB}$ via Eq. (S3) so that the right-hand side of the ODE system may be computed with $\underline{\hat{\beta}}$ in the appropriate range of values, allowing to obtain $\underline{\hat{y}}_j$.

$$\omega_k = \left(\frac{\hat{\beta}_k - LB_k}{UB_k - \hat{\beta}_k} \right)^{1/2} \tag{S2}$$

$$\frac{\hat{\beta}_k - LB_k}{UB_k - LB_k} = \frac{\omega_k^2}{\omega_k^2 + 1} \quad \Rightarrow \quad \hat{\beta}_k = LB_k + (UB_k - LB_k) \cdot \left(\frac{\omega_k^2}{\omega_k^2 + 1} \right) \qquad -\infty < \omega_k < +\infty \tag{S3}$$

Statistical Analysis

With assumptions (A1), (A2), (A3) and Eqs. (30)-(31) from the main text, as well as the numerically estimated parameters $\underline{\hat{\beta}}$ from the single-objective minimization (Eq. (32) from the main text), several statistical entities were calculated to check the goodness of the estimation and evaluate significance of parameters. The necessary statistical formulas are presented here with limited explanation (see Himmelblau, 1970). The main building block is the N_E *x* N_P Jacobian matrix $\underline{\underline{X}}_j$ of responses *j* relative to parameters, from which the N_P *x* N_P matrix $\left[\underline{\underline{XWX}} \right]$ is constructed as in Eq. (S4). Then the variance-covariance matrices of estimated parameters and predicted responses follow, respectively, in Eq. (S5).

$$\left[\underline{\underline{XWX}} \right] = \sum_{j=1}^{N_R} \left(\underline{\underline{X}}_j^T \underline{\underline{W}}_j \underline{\underline{X}}_j \right) \tag{S4}$$

$$\underline{\underline{Cov}}\left(\underline{\hat{\beta}}\right)=\sigma_{\varepsilon}^{2}\left[\underline{\underline{XWX}}\right]^{-1} \quad , \quad \underline{\underline{Cov}}\left(\underline{\hat{y}}_{j}\right)=\sigma_{\varepsilon}^{2}\underline{\underline{X}}_{j}\left[\underline{\underline{XWX}}\right]^{-1}\underline{\underline{X}}_{j}^{T} \tag{S5}$$

At the optimal $\underline{\hat{\beta}}$ solution, it can be shown (Himmelblau, 1970) that the statistic S_R^2 − the multi-response weighted sum of squared residuals given in Eq. (S6) − has several useful properties: *(i)* it corresponds to the optimum objective value in Eq. (32) (main text) divided by $N_R{\cdot}N_E - N_P$; *(ii)* it is uncorrelated with $\underline{\hat{\beta}}$; *(iii)* it is the best estimator (unbiased and coherent) of the fundamental variance σ_{ε}^2 ; and *(iv)* $S_R^2(N_R \cdot N_E - N_P)/\sigma_{\varepsilon}^2$ follows a chi-square (χ^2) PDF with $N_R \cdot N_E - N_P$ degrees of freedom. S_R^2 allows to obtain estimators of the variance-covariance matrices in Eq. (S5) as given in Eq. (S7). From Eq. (S7) one can extract estimators of standard deviations of estimated parameter $\hat{\beta}_k$ and estimated response *j* at experiment *i*, $\hat{y}_{j,i}$, as shown in Eq. (S8). Confidence intervals at 95% probability for true parameters and responses can be constructed as shown, respectively, in Eqs. (S9) and (S10), where $t_{1-\alpha/2}$ is the abscissa at $(1-\alpha/2)\cdot 100\%$ probability ($\alpha=0.05$) of the t-Student PDF with $N_R \cdot N_E - N_P$ degrees of freedom.

$$S_R^2=\left(\frac{1}{N_R \cdot N_E - N_P}\right)\sum_{j=1}^{N_R}\left(\underline{y}_j-\underline{\hat{y}}_j\right)^T\underline{\underline{W}}_j\left(\underline{y}_j-\underline{\hat{y}}_j\right) \tag{S6}$$

$$\underline{\underline{C\hat{o}v}}\left(\underline{\hat{\beta}}\right)=S_R^2\left[\underline{\underline{XWX}}\right]^{-1} \quad , \quad \underline{\underline{C\hat{o}v}}\left(\underline{\hat{y}}_{j}\right)=S_R^2\underline{\underline{X}}_{j}\left[\underline{\underline{XWX}}\right]^{-1}\underline{\underline{X}}_{j}^{T} \tag{S7}$$

$$\hat{\sigma}_{\hat{\beta}_k}=\sqrt{\left[\underline{\underline{C\hat{o}v}}(\underline{\hat{\beta}})\right]_{kk}} \quad , \quad \hat{\sigma}_{\hat{y}_{j,i}}=\sqrt{\left[\underline{\underline{C\hat{o}v}}(\underline{\hat{y}}_j)\right]_{ii}} \tag{S8}$$

$$\hat{\beta}_k - t_{1-\alpha/2}\cdot\hat{\sigma}_{\hat{\beta}_k} \;\le\; \beta_k \;\le\; \hat{\beta}_k + t_{1-\alpha/2}\cdot\hat{\sigma}_{\hat{\beta}_k} \tag{S9}$$

$$\hat{y}_{j,i} - t_{1-\alpha/2}\cdot\hat{\sigma}_{\hat{y}_{j,i}} \;\le\; \eta_{j,i} \;\le\; \hat{y}_{j,i} + t_{1-\alpha/2}\cdot\hat{\sigma}_{\hat{y}_{j,i}} \tag{S10}$$

The F-test to reject the null hypothesis (i.e. parameter β_k is significant) with 95% probability is given (Himmelblau, 1970) in Eq. (S11), where $\phi_{1-\alpha}\left(1, N_R \cdot N_E - N_P\right)$ is the abscissa at $(1-\alpha)\cdot 100\%$ probability ($\alpha=0.05$) of the Fisher PDF with degrees of freedom $(1, N_R \cdot N_E - N_P)$.

$$R(\hat{\beta}_k)=\frac{\left(\hat{\beta}_k\right)^2}{\left(\hat{\sigma}_{\hat{\beta}_k}\right)^2} > \phi_{1-\alpha}\left(1, N_R \cdot N_E - N_P\right) \;\Rightarrow\; \beta_k \neq 0 \tag{S11}$$

Table S4. F-test score $R_{\hat{\beta}_k}$ for the parameter estimates $\hat{\beta}_k$ with 95% probability.

Parameter	C1	C2	C3-A	C3-B	C3-C
$\phi_{1-\alpha}$ †	3.93	3.90	4.10	4.10	4.10
$v_{\max,CO}$	297	181	208	118	793
$v_{\max,H_2}$	140	54.3	247	222	696
$K_{S,CO}$	1293	13.0	188	67.8	306
K_{S,H_2}	246	32.3	396	54.1	1163
$K_{I,EtOH}$	123	-	-	-	-
$K_{I,HAc}$	226	10.3	228	427	92.6
$K_{I,CO}$	145	5.98	230	87.4	232
$Y_{X,CO}$	126	137	88.3	70.6	265
Y_{X,H_2}	293	24.5	158	185	1733
$v_{\max,CO}^{AcR}$	285	17.7	431	424	788
$K_{S,CO}^{AcR}$	1428	13.5	790	44.3	293
$v_{\max,H_2}^{AcR}$	444	33.2	352	90.8	780
K_{S,H_2}^{AcR}	613	16.3	487	137	351
k_d	2169	14.1	323	69.3	143
f_0	1786	31.1	414	65.5	263

† $R_{\hat{\beta}_k} > \phi_{1-\alpha}$ for parameter significance where $\phi_{1-\alpha}$ is the Fischer abscissa at 95% probability (α = 0.05) for degrees of freedom 1 and $N_R \cdot N_E - N_P$.

Additional variables with time profile

Fig. S1 presents the profiles of variables that are model inputs but also change with time, for case study C1. The agitation rate (*N*), inlet volumetric gas flow rate ($Q_{G,in}$) and volumetric liquid flow rate ($Q_{L,in}$) were obtained assuming linear profiles with time given the information provided in the article used as reference; and the mass transfer coefficient $k_L a_{ref}$ was calculated as explained in the main text. The liquid volume is 1000 mL.

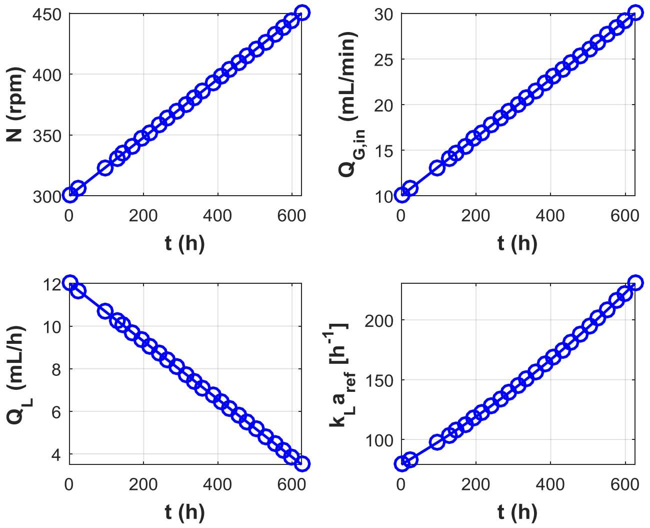

Figure S1. Dynamic profiles of agitation rate (*N*), inlet volumetric gas flow rate ($Q_{G,in}$), volumetric liquid flow rate ($Q_{L,in}$) and calculated $k_L a_{ref}$ in case study C1.

Effects of the initial conditions on the steady-state

(A) (B)

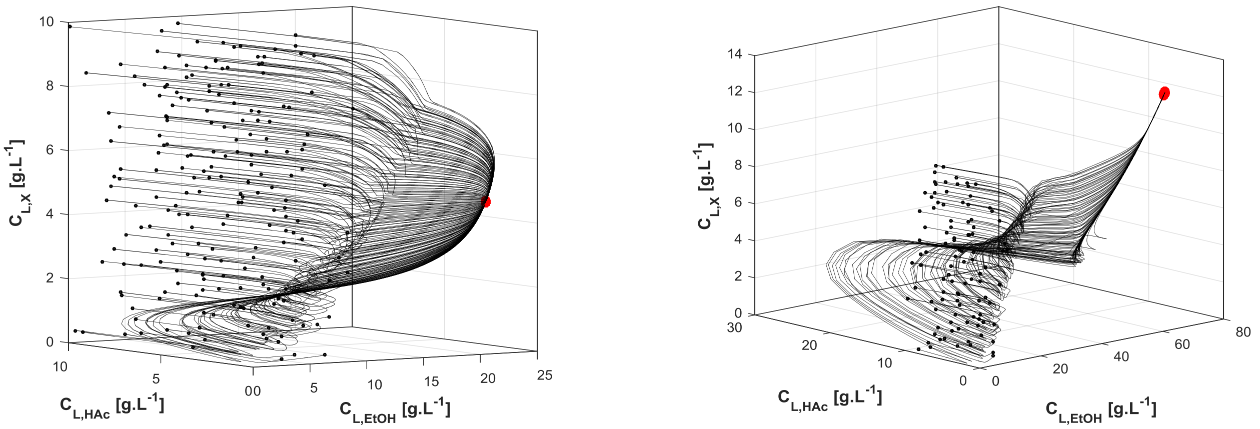

Figure S2. Phase portraits of ethanol, acetic acid and biomass concentrations with random perturbations on the initial conditions: dynamic trajectories (black solid lines), initial conditions (black spheres), steady states (red spheres). Operating conditions fixed at: (A) *GRT* = 50 min and D_{rate} = 0.01 h^{-1}; (B) *GRT* = 3.33 min and D_{rate} = 0.01 h^{-1}.

Effects of the agitation rate

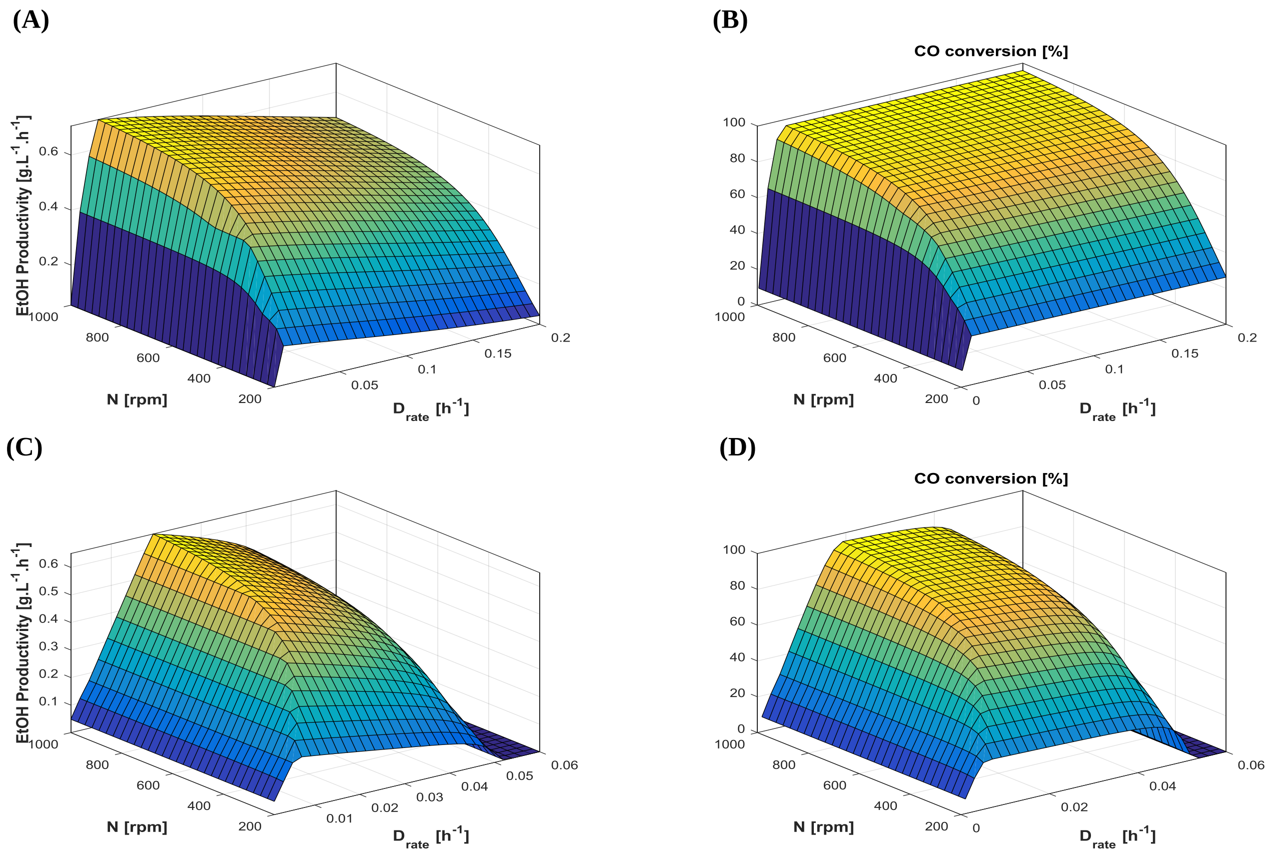

Figure S3. Steady-state ethanol productivity and CO conversion as function of agitation rate (N) and liquid dilution rate (D_{rate}): (A, B) with 90% cell recycle; (C, D) without cell recycle. Fixed conditions: y_{CO} = 0.65, y_{H_2} = 0.2, y_{CO_2} = 0.15, GRT = 20 min.

Effects of dilution rate and gas residence time with H_2-rich gas

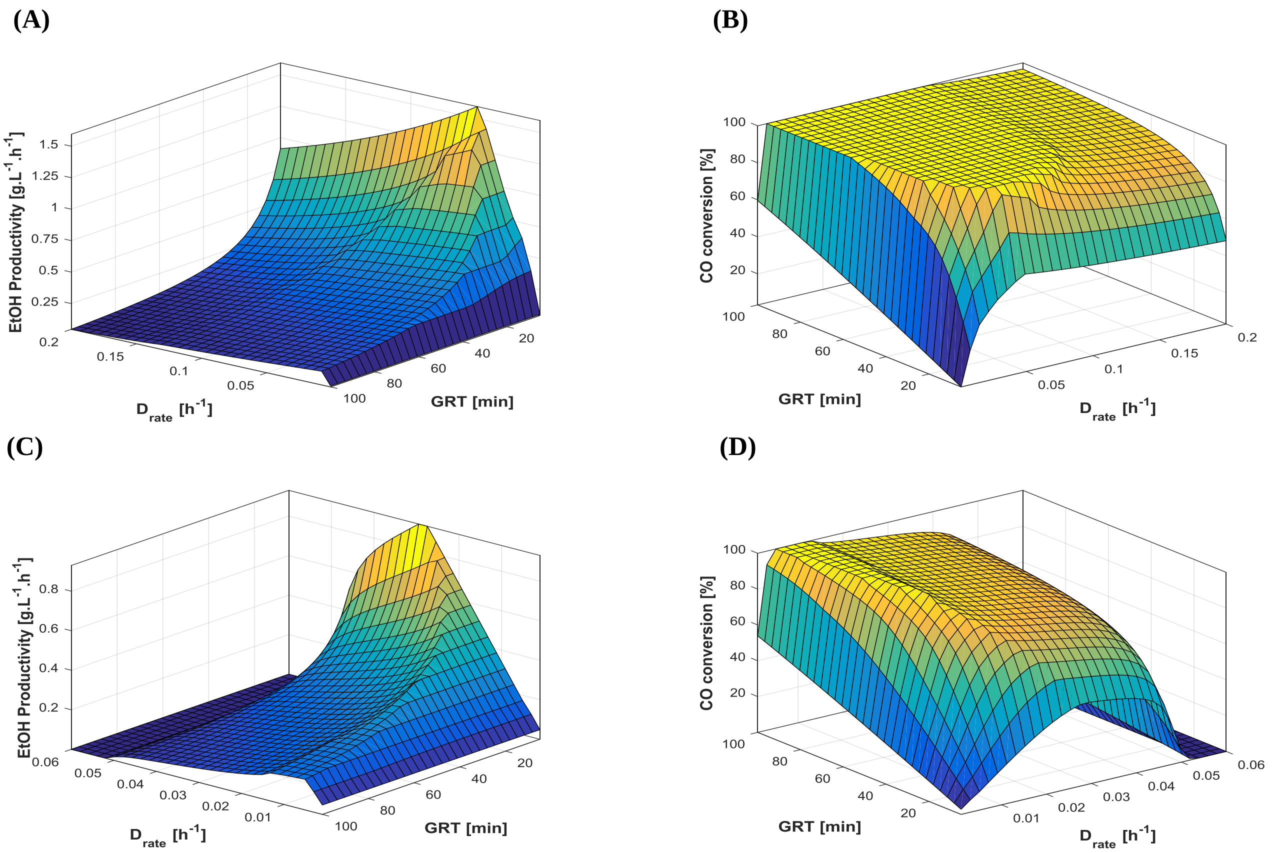

Figure S4. Steady-state ethanol productivity and CO conversion as function of gas residence time (*GRT*) and liquid dilution rate (D_{rate}): (A, B) with 90% cell recycle; (C, D) without cell recycle. Fixed conditions: $y_{CO} = 0.45$, $y_{H_2} = 0.5$, $y_{CO_2} = 0.05$, $N = 500$ rpm. Note: the axes are rotated in (A) and (C).

Optimization of process conditions and kinetic parameters

Table S5. Minimum and maximum values of the decision variables at the Pareto-optimal solutions

Decision Variable	1st run (65% CO, 20% H_2, 15% CO_2)		2nd run † (45% CO, 50% H_2, 5% CO_2)		3rd run †	
	min	max	min	max	min	max
GRT	5.45	49.8	8.22	32.7	7.79	29.9
D_{rate}	0.0396	0.173	0.0456	0.0607	0.0465	0.0605
XP	0.130	0.150	0.122	0.189	0.105	0.351
H_2:CO	-	-	-	-	0.778	0.855
$v_{\max,CO}$	42.8	45.3	43.7	45.0	38.5	43.2
$v_{\max,H_2}$	34.3	36.6	33.3	36.1	32.4	36.1
$Y_{X,CO}$	2.15	2.49	2.12	2.40	2.02	2.40
Y_{X,H_2}	0.200	0.299	0.209	0.267	0.196	0.289
$v_{\max,CO}^{AcR}$	39.0	42.1	31.8	33.3	33.9	35.4
$v_{\max,H_2}^{AcR}$	1.16	2.26	16.2	23.5	12.1	17.5
$K_{S,CO}^{AcR}$	362	365	398	428	397	404
K_{S,H_2}^{AcR}	471	488	403	485	358	449
k_d	0.00637	0.00742	0.00564	0.00870	0.00518	0.00878

† All points achieved 100% CO conversion.

Table S6. Selected solutions from the multi-objective optimization: 1st run (CO-rich gas)

GRT	D_{rate}	XP	$v_{\max,CO}$	$v_{\max,H_2}$	$Y_{X,CO}$	Y_{X,H_2}	$v_{\max,H_2}^{AcR}$	k_d	EtOH Productivity	CO conversion
49.8	0.173	0.130	45.3	36.6	2.44	0.273	1.54	0.00742	0.180	0.949
45.8	0.0505	0.133	44.4	35.7	2.43	0.259	1.49	0.00722	0.281	0.935
33.6	0.0574	0.133	43.9	35.8	2.42	0.239	1.51	0.00669	0.376	0.912
25.2	0.0407	0.133	43.6	35.9	2.38	0.232	1.45	0.00675	0.506	0.883
20.9	0.0404	0.133	43.6	35.7	2.40	0.258	1.40	0.00644	0.598	0.860
16.5	0.0404	0.137	43.6	35.5	2.41	0.226	1.21	0.00652	0.730	0.824
14.1	0.0438	0.134	43.8	35.0	2.39	0.220	1.20	0.00644	0.821	0.797
11.5	0.0401	0.134	43.4	35.4	2.41	0.219	1.31	0.00642	0.963	0.754
9.75	0.0397	0.134	43.6	35.5	2.40	0.239	1.24	0.00648	1.08	0.716
7.80	0.0417	0.133	43.7	34.4	2.38	0.223	1.82	0.00643	1.23	0.655
6.69	0.0411	0.134	43.4	34.8	2.41	0.211	1.31	0.00644	1.35	0.613
5.71	0.0490	0.134	43.7	35.5	2.30	0.205	1.45	0.00650	1.45	0.564

Table S7. Selected solutions from the multi-objective optimization: 2nd run (H_2-rich gas)

GRT	D_{rate}	XP	$v_{\max,CO}$	$v_{\max,H_2}$	$Y_{X,CO}$	Y_{X,H_2}	$v^{AcR}_{\max,H_2}$	k_d	EtOH Productivity	CO conversion
32.7	0.0519	0.165	44.4	33.9	2.30	0.236	22.4	0.00770	0.481	1.00
29.6	0.0505	0.149	44.4	33.9	2.29	0.230	21.7	0.00766	0.543	1.00
24.8	0.0535	0.154	44.3	34.0	2.28	0.228	21.4	0.00833	0.639	1.00
22.7	0.0534	0.154	44.3	34.5	2.29	0.227	21.1	0.00823	0.704	1.00
19.0	0.0527	0.153	44.1	34.0	2.30	0.232	21.3	0.00779	0.849	1.00
17.1	0.0537	0.161	44.2	34.0	2.30	0.231	20.3	0.00802	0.948	1.00
14.6	0.0607	0.151	44.2	34.1	2.22	0.231	23.5	0.00734	1.11	1.00
10.1	0.0534	0.125	43.8	34.2	2.26	0.215	19.7	0.00684	1.64	1.00
9.32	0.0566	0.123	44.1	34.6	2.32	0.236	18.2	0.00670	1.77	1.00
9.05	0.0562	0.124	44.4	35.9	2.35	0.249	18.7	0.00670	1.83	1.00
8.75	0.0559	0.123	44.3	35.3	2.39	0.251	17.3	0.00657	1.88	1.00
8.52	0.0570	0.123	44.7	35.8	2.39	0.258	17.1	0.00575	1.93	0.997

Table S8. Selected solutions from the multi-objective optimization: 3nd run

GRT	D_{rate}	XP	H_2:CO	$v_{\max,CO}$	$v_{\max,H_2}$	$Y_{X,CO}$	Y_{X,H_2}	$v^{AcR}_{\max,H_2}$	k_d	EtOH Productivity	CO conversion
29.9	0.0465	0.351	0.822	41.0	34.2	2.02	0.208	17.5	0.00711	0.543	1.00
15.7	0.0517	0.217	0.833	40.8	32.4	2.18	0.210	16.1	0.00668	1.09	1.00
13.2	0.0497	0.218	0.855	39.4	35.8	2.36	0.225	17.3	0.00831	1.31	1.00
10.4	0.0542	0.160	0.842	40.2	34.2	2.11	0.223	17.2	0.00799	1.67	1.00
9.86	0.0528	0.174	0.840	40.0	34.2	2.30	0.210	16.8	0.00692	1.78	1.00
9.54	0.0572	0.136	0.836	40.5	33.8	2.20	0.220	16.5	0.00553	1.83	1.00
9.25	0.0568	0.139	0.844	40.9	34.0	2.20	0.223	16.5	0.00559	1.89	1.00
9.07	0.0580	0.137	0.847	40.4	34.1	2.21	0.221	16.3	0.00529	1.92	1.00
8.92	0.0584	0.127	0.850	40.2	34.6	2.26	0.219	16.4	0.00555	1.95	1.00
8.76	0.0591	0.118	0.849	40.3	35.0	2.36	0.223	16.8	0.00542	2.00	1.00
8.18	0.0603	0.118	0.850	40.4	34.8	2.36	0.246	16.7	0.00546	2.03	0.952
7.79	0.0605	0.116	0.852	40.3	35.5	2.21	0.288	16.6	0.00546	2.04	0.915

Cell mass concentration

Figures S5-S8 show the ranges of cell mass concentration that correspond to the conditions presented in Figs. 5-8 from the main text.

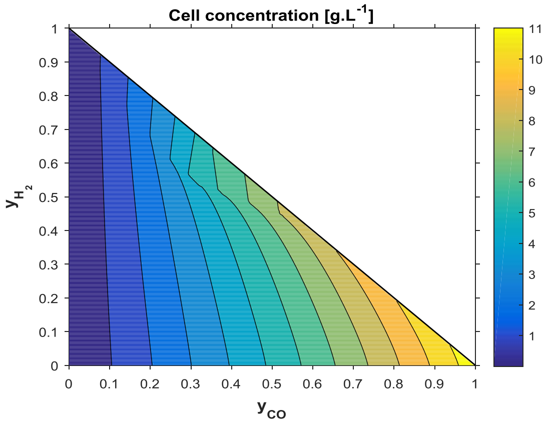

Figure S5. Predicted steady-state cell concentration achieved with different gas compositions $(y_{CO_2} = 1 - y_{CO} - y_{H_2})$, with fixed conditions: *GRT* = 20 min, D_{rate} = 0.025 h^{-1}, *N* = 500 rpm, cell recycle = 90%.

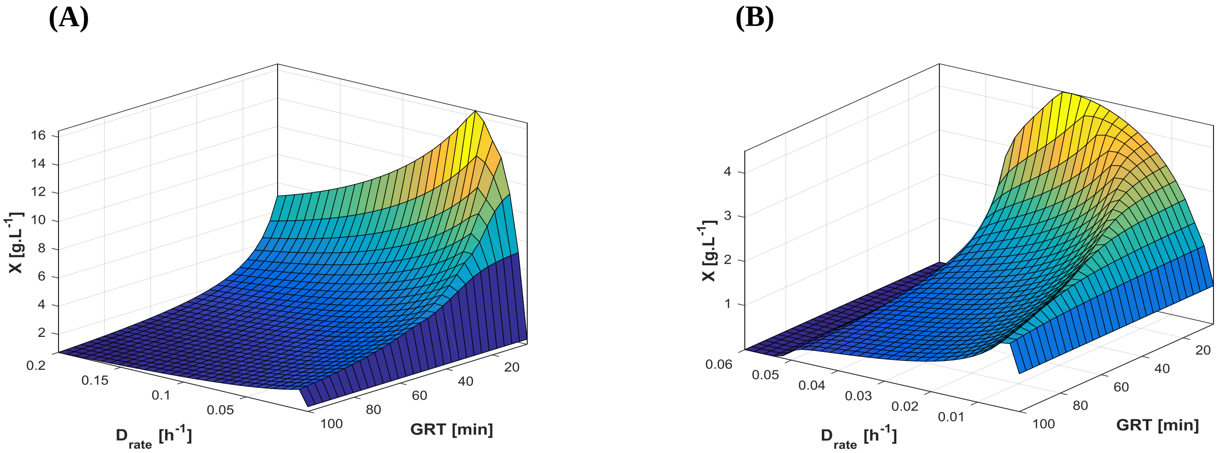

Figure S6. Steady-state cell mass concentration as function of gas residence time (*GRT*) and liquid dilution rate (D_{rate}): (A) with 90% cell recycle; (B) without cell recycle. Fixed conditions: $y_{CO} = 0.65$, $y_{H_2} = 0.2$, $y_{CO_2} = 0.15$ and *N* = 500 rpm.

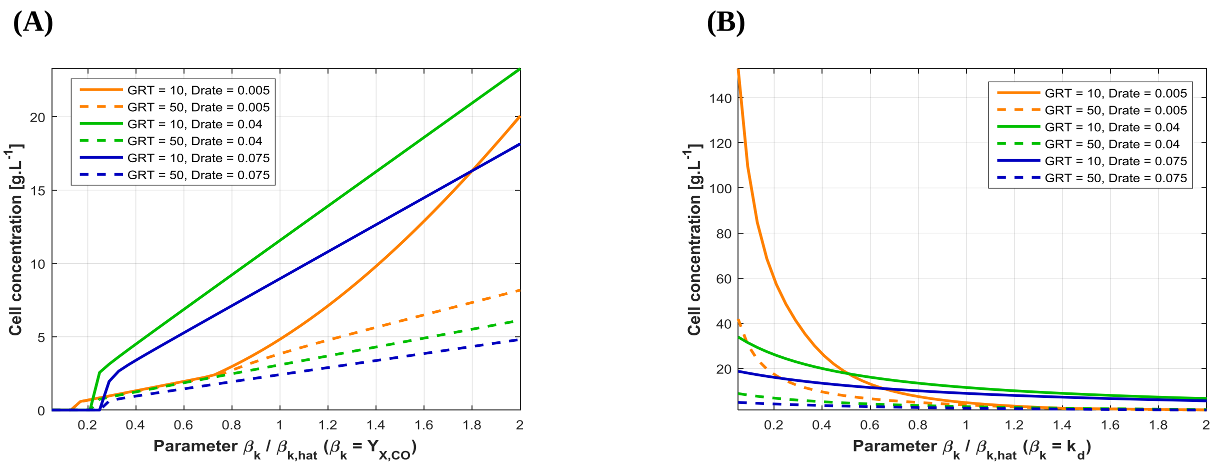

Figure S7. Sensitivity of steady-state cell mass concentration to kinetic parameters under different conditions of gas residence time (*GRT*) and liquid dilution rate (D_{rate}): (A) cell yield on CO ($Y_{X/CO}$); (B) cell death rate constant (k_d). Fixed conditions: $y_{CO} = 0.65$, $y_{H_2} = 0.2$, $y_{CO_2} = 0.15$ and N = 500 rpm.

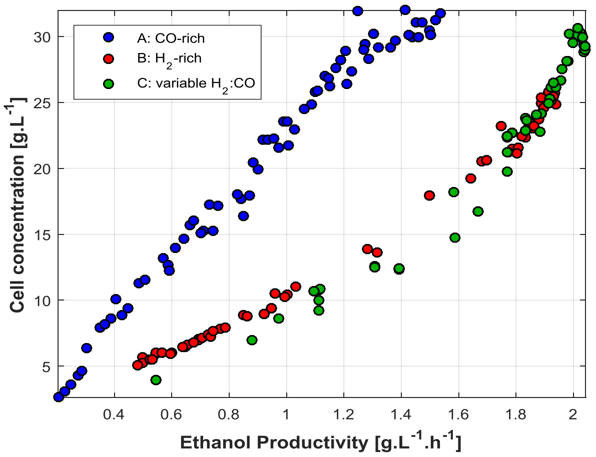

Figure S8. Values of cell mass concentration at the Pareto-optimal solutions obtained for maximization of steady-state ethanol productivity and CO conversion (see Fig. 8 of the main text). Colors indicate the optimization runs: (A) 1st run, with fixed CO-rich gas composition; (B) 2nd run, with fixed H_2-rich gas composition; (C) 3rd run, with variable gas composition.

References for the Supplementary Materials

Cussler, E. L. (1997). *Diffusion: Mass Transfer in Fluid Systems* (2nd ed.). New York: Cambridge University Press.

Himmelblau, D. M. (1970). *Process analysis by statistical methods*. New York: John Wiley & Sons, Inc.

Perry, R. H., Green, D. W. (1999). *Perry's chemical engineers' handbook* (7th ed.). McGraw-Hill.

Sander, R. (1999). *Compilation of Henry's law constants for inorganic and organic species of potential importance in environmental chemistry*.
